# Comparative safety profiles of first-line immunotherapy regimens in advanced esophageal squamous cell carcinoma: a network meta-analysis focusing on toxicity stratification

**DOI:** 10.3389/fonc.2025.1700236

**Published:** 2026-01-02

**Authors:** Wei Chen, Boyan Chen, Chunbin Hu, Qiance Wei, Jiayi Chen, Wenxin Xue, Shui Liu, Lichaoyue Sun, Lili Zhang, Shuo Fan

**Affiliations:** 1 Department of Pharmacy, Emergency General Hospital, Beijing, China; 2 First Clinical Medical College, Nanjing university of Chinese medicine, Beijing, China; 3 School of Basic Medical Sciences, Capital Medical University, Beijing, China; 4 School of Nursing, Zhejiang University, Hangzhou, Zhejiang, China; 5 Pharmacy Department, Aerospace Center Hospital, Beijing, China

**Keywords:** ESCC, ICIS, immune-related adverse events, network meta-analysis, safety

## Abstract

**Background:**

The combination of immune checkpoint blockade and chemotherapy significantly improves survival when used as first-line treatment for advanced esophageal squamous cell carcinoma. Nonetheless, the safety profiles of various immune checkpoint inhibitor (ICI)-based combination therapies remain inadequately characterized, particularly regarding the incidence of severe adverse events (AEs) and immune-related adverse events (irAEs). The research employed a network meta-analysis to systematically evaluate and contrast the toxicity profiles across various initial ICI-driven treatments for advanced Esophageal squamous cell carcinoma (ESCC).

**Methods:**

An extensive literature review was conducted across PubMed, EMBASE, the Cochrane Library, and Web of Science to identify RCTs evaluating first-line immunotherapy in advanced ESCC. The search included all records from each database’s inception to July 1, 2025. Primary endpoints included grade ≥3 treatment-related adverse events(grade ≥3 trAEs), any-grade irAEs, and grade ≥3 irAEs. Secondary analyses focused on organ-specific irAEs, including immune-mediated rash, hypothyroidism, hyperthyroidism, and pneumonitis. We conducted a Bayesian network meta-analysis to assess relative risks (RRs) and establish a treatment ranking based on Surface Under the Cumulative Ranking Curve (SUCRA) metrics, evaluating the comparative effectiveness of different therapeutic options. The study protocol was prospectively registered with PROSPERO (CRD420251113069).

**Results:**

Seven randomized controlled trials involving 4,479 patients with advanced ESCC were included. In pairwise meta-analyses, ICI plus chemotherapy, compared with chemotherapy alone, increased the risk of grade ≥3 treatment-related adverse events (RR 1.08, 95% CI 1.00-1.17), any-grade irAEs (RR 2.04, 95% CI 1.71-2.44), and grade ≥3 irAEs (RR 2.75, 95% CI 1.98-3.82). Immune-chemotherapy also significantly elevated the risks of immune-mediated rash, hypothyroidism, and hyperthyroidism, whereas the increase in immune-mediated pneumonitis did not reach statistical significance. In Bayesian network meta-analyses, camrelizumab plus chemotherapy had the highest probability of being the regimen with the lowest risk of grade ≥3 trAEs(SCURA = 87.8%) and grade ≥3 irAEs(SCURA = 71.6%), while toripalimab plus chemotherapy ranked safest for any-grade irAEs(SCURA = 83.8%).

**Conclusions:**

First-line ICI-based regimens for advanced ESCC are associated with an increased risk of severe treatment-related and immune-related toxicities compared with chemotherapy alone, and their safety profiles differ substantially across regimens. Our SUCRA-based rankings provide a comparative overview of regimen-level toxicity that may assist clinicians in understanding relative safety trade-offs when selecting first-line therapy. These findings should be integrated with regimen-specific efficacy data, regulatory indications, PD-L1 status, comorbidities, and patient preferences, and are best interpreted as complementary safety evidence rather than stand-alone treatment recommendations.

**Systematic Review Registration:**

https://www.crd.york.ac.uk/prospero/#myprospero, identifier CRD420251113069.

## 1 Introduction

Esophageal cancer is the seventh leading cause of cancer mortality worldwide, with more than 510,700 new cases and over 445,000 deaths each year. ESCC predominates in Asia and Africa and accounts for approximately 84% of global esophageal cancer cases (1). Despite the gradual expansion of screening programs, over half of patients present with advanced disease, and outcomes remain poor: the overall 5-year survival rate is below 20% (2).

Historically, platinum-based combination chemotherapy has been the first-line treatment for advanced ESCC. However, despite its broad use, its benefit has been limited, with median overall survival typically under 10 months. Additionally, treatment is often accompanied by significant toxicities that severely affect quality of life (3, 4). This has underscored a critical challenge in oncology: while efficacy remains paramount, the toxicity burden of treatment is a major determinant of patient outcomes and quality of life. Thus, understanding and managing irAEs have become central to treatment decisions, especially in the context of immunotherapies that have reshaped the therapeutic landscape.

Immunotherapy, particularly ICIs, has dramatically altered the treatment paradigm for advanced cancers, including ESCC (5, 6). By blocking inhibitory pathways such as PD-1, PD-L1, or CTLA-4, ICIs reinvigorate T-cell-mediated antitumor immunity and can produce durable clinical remissions (7, 8). This evolution in cancer treatment has marked a shift from cytotoxic therapies to immune modulation. However, while the clinical benefits of ICIs are substantial, these therapies are also associated with a distinct toxicity profile—irAEs—which can affect various organs, including the skin, lungs, liver, endocrine organs, and gastrointestinal tract. Severe irAEs may even be life-threatening (9, 10). The introduction of ICI-based regimens for advanced ESCC has raised important questions regarding the balance of efficacy and toxicity. Although several randomized trials, such as KEYNOTE-590, ESCORT-1st, and ORIENT-15, have shown that the addition of PD-1 inhibitors to chemotherapy improves survival outcomes, these studies have predominantly focused on efficacy endpoints, leaving the nuanced safety profiles of these regimens underexplored (11–13). In particular, the frequency and severity of irAEs vary significantly across different ICI-based regimens, which are often used in combination with chemotherapy (14). Understanding how these toxicity profiles influence treatment decisions is crucial, not only for clinicians but also for the multidisciplinary teams involved in managing ESCC patients (15). It is critical to consider how irAEs impact patient quality of life, requiring careful management and potential adjustments to treatment regimens.

Although several head-to-head randomized trials have evaluated the efficacy of ICI-chemotherapy in advanced ESCC, most have prioritized survival endpoints, and comprehensive, regimen-level safety comparisons—particularly of immune-mediated toxicities—remain limited. Differences in molecular structure, target affinity, and immunogenicity among ICIs may drive substantial variation in both the incidence and severity of adverse events (16).Moreover, because most trials juxtapose a single ICI-based regimen with chemotherapy, direct comparisons among competing ICI combinations are scarce, leaving clinicians to rely on indirect evidence or isolated study results rather than a unified evidence base.

To further explore these complexities, we conducted a systematic review and Bayesian network meta-analysis (NMA) to compare the safety of various first-line ICI-based regimens for advanced ESCC. By synthesizing data on grade ≥3 treatment-related adverse events(Grade≥3 trAEs), all-grade irAEs, and organ-specific toxicities (e.g., rash, thyroid dysfunction, and immune-mediated pneumonitis), we aimed to delineate the relative safety profiles across regimens. This analysis provides decision-ready, evidence-based guidance for clinicians to optimize treatment selection, taking into account both efficacy and toxicity, and supports the broader goal of personalized, multidisciplinary care.

## 2 Materials and methods

This NMA was conducted in accordance with the PRISMA extension for network meta-analyses (PRISMA-NMA; **Supplementary Table S1**) (17).Owing to the paucity of head-to-head randomized trials comparing alternative immunotherapy-based regimens, we adopted a Bayesian framework to enable indirect comparisons and to generate probabilistic estimates of safety and toxicity (18).The study protocol was prospectively registered with the International Prospective Register of Systematic Reviews (CRD420251113069) to ensure transparency, methodological rigor, and reproducibility.

### 2.1 Literature retrieval and search methodology

We systematically searched PubMed, Embase, the Cochrane Library, and the Web of Science Core Collection from database inception to July 1, 2025. The search strategy combined controlled vocabulary (e.g., MeSH/Emtree) with free-text terms using Boolean operators. Core terms encompassed the disease (“esophageal squamous cell carcinoma” OR “oesophageal squamous cell carcinoma”), study design (“randomized controlled trial” OR “randomized clinical trial” OR “RCT”), and immunotherapy (“immune checkpoint inhibitor” OR “PD-1 inhibitor” OR “PD-L1 inhibitor” OR “CTLA-4 inhibitor”), as well as specific agents (sintilimab, pembrolizumab, toripalimab, camrelizumab, nivolumab, ipilimumab, serplulimab, sugemalimab) (**Supplementary Table S2**). The complete, database-specific search strings are provided in **Supplementary Table S2**.

### 2.2 Eligibility criteria and study selection process

Inclusion Criteria:

1. Design: Randomized controlled trials enrolling patients with histologically or cytologically confirmed advanced or metastatic ESCC.Patients aged ≥18 years with unresectable, recurrent, locally advanced, or metastatic ESCC. Trials with mixed histologies were eligible only if ESCC subgroup safety data were extractable.
2. Intervention: RCTs assessing first-line immune checkpoint inhibitor therapy, either as monotherapy or in combination with chemotherapy.
3. Comparator: Control arms comprised either chemotherapy alone or single-agent immune checkpoint inhibitors, both delivered as first-line regimens.
4. Safety Outcomes: Studies that provided safety outcome data, specifically including: Grade≥3 trAEs, defined as the proportion of patients who experienced at least one (Common Terminology Criteria for Adverse Events) CTCAE Grade 3–5 adverse event considered by the investigators to be related to study treatment during the on-treatment period and follow-up safety window.; as well as any-grade irAEs affecting major organ systems, such as the skin, endocrine glands, lungs, liver, or gastrointestinal tract; Grade ≥3 irAEs; Organ-specific immune-mediated toxicities, including rash, hypothyroidism, hyperthyroidism, and pneumonitis. Diagnosis of irAEs was required to be based on comprehensive clinical evaluation, relevant laboratory and radiologic findings, and the exclusion of alternative etiologies.

Exclusion Criteria:

1. RCTs describing different treatment phases or sub-analyses based on overlapping patient cohorts.
2. Studies lacking clear or complete reporting of safety endpoints.
3. Publications not presenting original research, including reviews, editorials, commentaries, and case reports, were excluded.

Two independent reviewers carried out the selection process, with all included trials cross-checked to confirm data currency and to avoid duplication.

### 2.3 Definition and harmonization of safety endpoints

To ensure comparability of safety endpoints across trials, we prospectively harmonised adverse event definitions before data extraction. Because the seven included randomised controlled trials used different CTCAE versions (v4.0, v4.03, or v5.0) and reported heterogeneous safety datasets, we first identified, for each study, the source dataset for grade ≥3 events. All trials reported treatment-related adverse events; accordingly, we operationally defined grade ≥3 trAEs as any CTCAE grade 3–5 treatment-related adverse event and extracted event counts directly from the trial-specific grade ≥3 treatment-related AE tables.

IrAEs were extracted strictly according to the original trial definitions. In all studies, immune-mediated toxicities were classified using sponsor- or protocol-prespecified MedDRA term lists and were reported by investigators under blinded conditions; in CheckMate 648, these immune-mediated events were additionally subjected to *post hoc* adjudication by the sponsor. To avoid introducing classification bias, we adhered fully to the trial-defined immune-mediated categories, did not reassign events that were not labelled as immune-mediated in the original reports, and did not expand or modify the sponsor-specified groupings.

The selection of organ-specific irAEs was based on two considerations. First, these toxicities are among the most frequent and clinically relevant immune-mediated events in the context of anti-PD-1/PD-L1 therapy. Second, only immune-mediated rash, hypothyroidism, hyperthyroidism, and pneumonitis were reported with sufficiently consistent numerical data across the majority of trials. Consequently, quantitative analyses were restricted to these four organ-specific irAEs. When a trial did not provide explicit numerical data for a given organ-specific irAE, that study was excluded from the corresponding endpoint analysis and missing data were not imputed as zero events.

**Supplementary Table S8** summarises, for each trial, the CTCAE version used, the type of safety dataset, the operational definition and ascertainment of immune-mediated AEs, and the availability of organ-specific irAE data, thereby facilitating cross-trial comparison and methodological reproducibility.

### 2.4 Data collection and risk of bias assessment

Three reviewers independently extracted data from eligible randomized controlled trials in accordance with PRISMA guidance; disagreements were resolved through discussion with a fourth reviewer. From each study, we recorded the trial name; design and randomization ratio; publication source and year; trial phase; tumor stage; trial registration; sample size; patient age and sex distributions; and details of interventions and comparators.

Risk of bias for included RCTs was appraised using the Cochrane Risk of Bias 2.0 tool (19), which evaluates five domains: bias arising from the randomization process; bias due to deviations from intended interventions; bias due to missing outcome data; bias in measurement of the outcome; and bias in selection of the reported result. Overall judgments were categorized as “low risk,” “some concerns,” or “high risk.”&.

### 2.5 Statistical methods and analytical framework

The primary outcomes were grade≥3 trAEs, any-grade irAEs, and grade ≥3 irAEs. Secondary outcomes comprised immune-mediated rash, immune-mediated hypothyroidism, immune-mediated hyperthyroidism, and immune-mediated pneumonitis. For irAEs and chemotherapy-related AEs, we extracted the relevant data from each study reported in the literature. In the case of immune-mediated events, the literature typically clearly identifies them as immune-mediated reactions, so we classified and recorded them based on the detailed descriptions provided in the studies. Dichotomous endpoints were synthesized as RRs with 95% CIs.We conducted a Bayesian network meta-analyses were conducted in R using the gemtc (version 4.4) and rjags packages. Both fixed-effect and random-effects models were fitted, and model selection was guided by the deviance information criterion (DIC). When the difference in DIC between the two models was less than 5, indicating no meaningful difference in model fit, the fixed-effect model was preferred for subsequent analyses. Posterior inference employed Markov chain Monte Carlo (MCMC) with four parallel chains, a burn-in of 20,000 iterations per chain, followed by 100,000 sampling iterations. Convergence was assessed by visual inspection of trace/diagnostic plots and the Gelman-Rubin statistic.To rank regimens across primary and secondary safety endpoints, we calculated the SUCRA; for safety outcomes, higher SUCRA values denote a more favorable (lower-risk) profile. Posterior rank probabilities (i.e., the probability that each treatment occupies ranks 1 through N) were obtained with rank.probability, and a heatmap was generated with pheatmap to visualize comparative rankings. Global consistency was examined by comparing DIC values from consistency and inconsistency models; ΔDIC >5 was interpreted as evidence of important inconsistency requiring further exploration.

Additionally, head-to-head comparisons of immunochemotherapy versus chemotherapy alone were synthesized under a frequentist framework in RevMan 5.4. Between-study heterogeneity was evaluated using Cochran’s Q and the *I²* statistic. When *I²* ≤50% or *P* ≥0.10 indicated low heterogeneity, a fixed-effect model was applied; otherwise, a random-effects model was used. For results with substantial heterogeneity, sensitivity analyses were performed by sequentially omitting studies contributing most to heterogeneity to test the robustness of pooled estimates. Small-study effects/publication bias were explored with funnel plots. Two-sided tests were used throughout, with α=0.05.

### 2.6 Certainty of evidence

We rated the certainty of evidence for pre-specified key outcomes using GRADE as high, moderate, low, or very low across five domains: risk of bias, inconsistency, indirectness, imprecision, and publication bias. A Summary of Findings (SoF) table was generated with GRADE, and relative effects were translated into absolute effects per 1,000 patients based on representative baseline risks observed in the control arms. Two reviewers independently performed the GRADE assessments, with disagreements resolved by adjudication from a third reviewer.

## 3 Results

### 3.1 Study selection and baseline characteristics of trials

**Figure 1:**
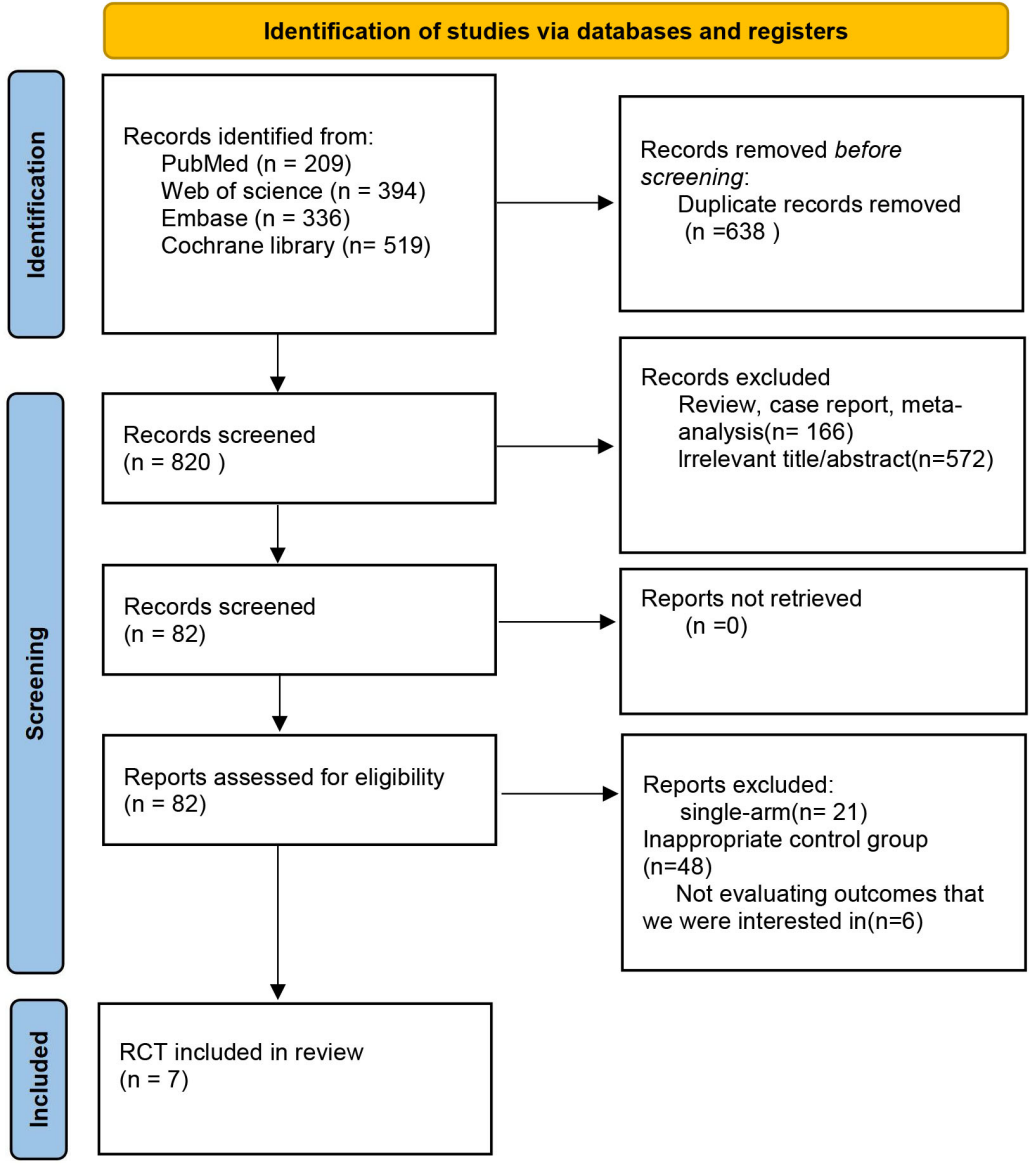
PRISMA diagram depicting study screening and inclusion process.

An initial pool of 1,458 records was retrieved via a comprehensive database search. After removing duplicates and screening titles and abstracts for relevance, 82 articles were selected for full-text evaluation. After thorough evaluation, seven randomized controlled trials satisfied the inclusion criteria and were incorporated into the final analysis, as shown in **Figure 1**.

Together, these seven trials enrolled 4,479 patients, who were treated with one of nine first-line regimens. These included sintilimab combined with chemotherapy (Sinti-chemo), tislelizumab combined with chemotherapy (Tisle-chemo), toripalimab combined with chemotherapy (Toripa-chemo), camrelizumab combined with chemotherapy (Camre-chemo), nivolumab combined with chemotherapy (Nivo-chemo), nivolumab combined with ipilimumab (Nivo-ipi), serplulimab combined with chemotherapy (Serplu-chemo), sugemalimab combined with chemotherapy (Sugema-chemo), and chemotherapy alone (Chemo). Comprehensive characteristics of the included trials, including study design, sample size, treatment regimens, and baseline demographic and clinical profiles, are systematically summarized in **Table 1**. To ensure reproducibility for readers, all extracted safety data are presented in **Supplementary Tables S9** and **Supplementary Tables S10**.

**Table 1:** Baseline features of the included clinical trials.

Study	Publication year	Trial registration ID	Stage	(Phase, design)	Median age/y	Male /female	Ethnicity	Intervention group	Control group
ORIENT-15 (13)	2022	NCT03748134	III-IV	double-blind III	63/63	567/92	Asian (97.3%) White (1.8%)	Sintilimab (3 mg/kg for body weight <60 kg or 200 mg for ≥60 kg, IV, Q3W) + cisplatin (75 mg/m², IV, Q3W) combined with either:paclitaxel (87.5 mg/m² on days 1 and 8 in cycle 1, then 175 mg/m² on day 1 from cycle 2 onward, Q3W), or 5-fluorouracil (800 mg/m², IV, Q3W).	Placebo (3 mg/kg for <60 kg or 200 mg for ≥60 kg, IV, Q3W) + cisplatin (75 mg/m², IV, Q3W) combined with either:paclitaxel (same schedule as above), or 5-fluorouracil (800 mg/m², IV, Q3W).
JUPITER-06 (20)	2022	NCT03829969	IV	double-blind III	63/62	437/77	China (100%)	Toripalimab 240 mg IV Q3W + paclitaxel 175 mg/m² IV Q3W + cisplatin 75 mg/m² IV Q3W.	Placebo (IV, Q3W) + paclitaxel 175 mg/m² IV Q3W + cisplatin 75 mg/m² IV Q3W.
ESCORT-1st (12)	2024	NCT03691090	IV	open-label III	62/62	523/73	China (100%)	Camrelizumab 200 mg IV Q3W + paclitaxel 175 mg/m² IV Q3W + cisplatin 75 mg/m² IV Q3W.	Placebo (IV, Q3W) + paclitaxel 175 mg/m² IV Q3W + cisplatin 75 mg/m² IV Q3W.
Checkmate 648 (21)	2022	NCT03143153	IV	open-label III	64/63/64	797/173	Asian (70.6%) White (25.6%) Black (1.1%)	Experimental group A (Nivolumab + Chemotherapy): Nivolumab 240 mg IV Q2W + 5-fluorouracil 800 mg/m²/day IV on days 1-5 + cisplatin 80 mg/m² IV on day 1, repeated every 2 weeks. Experimental group B (Nivolumab + Ipilimumab): Nivolumab 3 mg/kg IV Q2W + ipilimumab 1 mg/kg IV Q6W.	5-fluorouracil 800 mg/m²/day IV on days 1-5 + cisplatin 80 mg/m² IV on day 1, repeated every 2 weeks.
ASTRUM-007 (22)	2023	NCT03958890	III-IV	double-blind III	64/64	470/81	China (100%)	Serplulimab 3 mg/kg IV on day 1 + cisplatin 50 mg/m² IV on day 1 + 5-fluorouracil 1200 mg/m²/day IV on days 1-2, repeated every 2 weeks (Q2W).	Placebo (IV, day 1) + cisplatin 50 mg/m² IV on day 1 + 5-fluorouracil 1200 mg/m²/day IV on days 1-2, repeated every 2 weeks (Q2W).
GEMSTONE-304 (23)	2024	NCT04187352	III-IV	double-blind III	62.5/63	472/68	Asian(100%)	Sugemalimab 1200 mg IV Q3W + nab-paclitaxel 260 mg/m² IV Q3W + cisplatin 75 mg/m² IV Q3W for up to 6 cycles, followed by sugemalimab monotherapy maintenance until progression or unacceptable toxicity.	Placebo (IV, Q3W) + nab-paclitaxel 260 mg/m² IV Q3W + cisplatin 75 mg/m² IV Q3W for up to 6 cycles, followed by placebo maintenance.
RATIONALE-306 (24)	2023	NCT03783442	III-IV	double-blind III	64/65	563/86	Asian (74.9%) White (23.9%)	Tislelizumab 200 mg IV Q3W plus investigator-chosen platinum doublet (cisplatin or oxaliplatin combined with fluorouracil, capecitabine, or paclitaxel) for up to 6 cycles, with tislelizumab continued for up to 2 years or until disease progression or unacceptable toxicity.	Placebo IV Q3W plus investigator-chosen platinum-based chemotherapy (cisplatin or oxaliplatin combined with fluorouracil, capecitabine, or paclitaxel) for up to 6 cycles, followed by placebo maintenance.

### 3.2 Risk of bias assessment

Among the seven included trials, four were judged to have an overall low risk of bias, whereas three were rated as raising some concerns. With respect to the randomization process, all studies were deemed at low risk, having employed appropriate random sequence generation methods and reasonable stratification strategies. Because ESCORT-1st and CheckMate 648 used open-label designs, knowledge of treatment allocation could, in principle, have influenced co-interventions or other behaviors; therefore, the domain “deviations from intended interventions” was rated as some concerns, despite the largely objective nature of the primary endpoints (OS/PFS).

**Figure 2:**
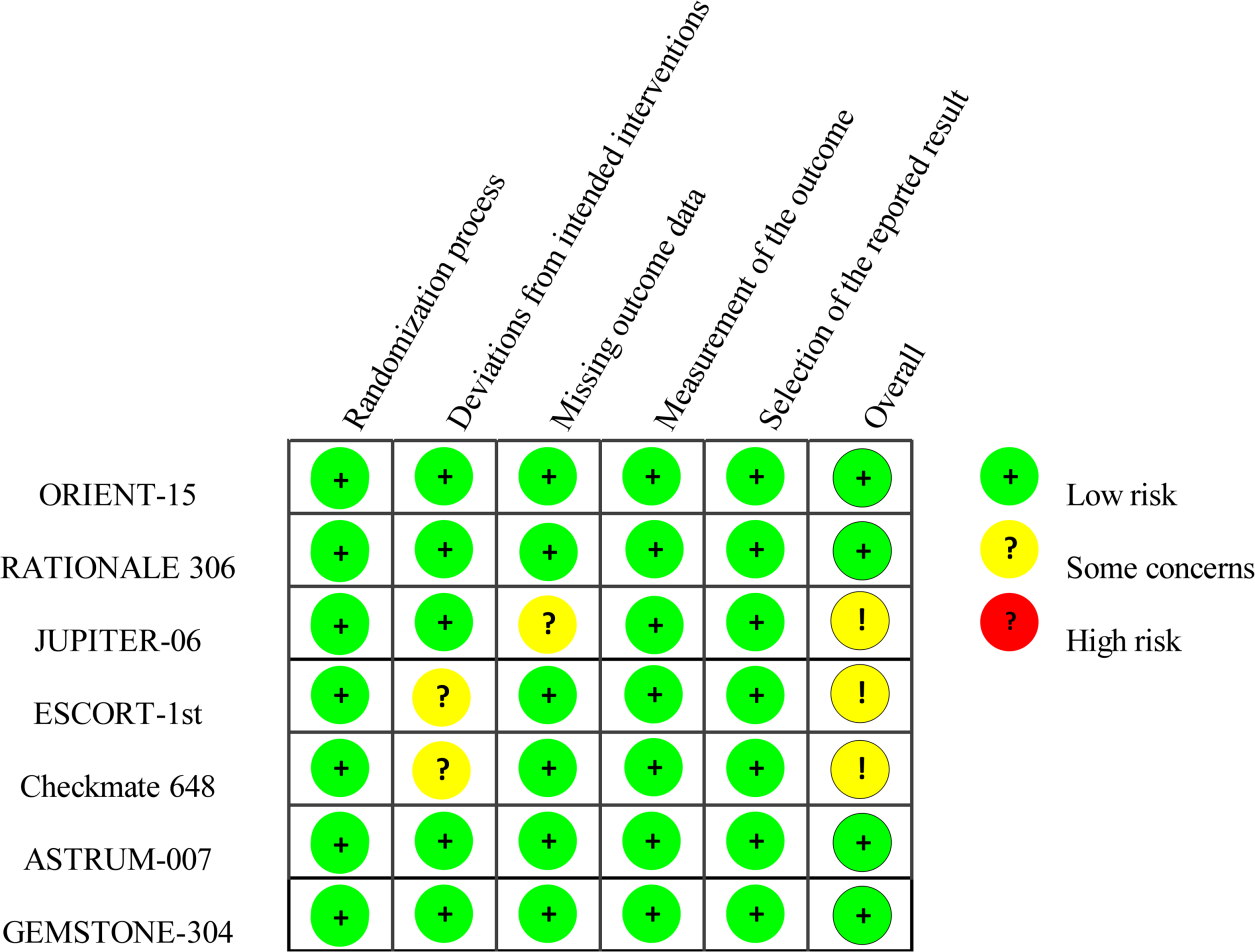
Overview of risk of bias assessment in included RCTs based on the RoB 2 tool.

For missing outcome data, JUPITER-06 was rated some concerns due to interruptions in follow-up for a subset of participants, whereas the remaining trials conducted intention-to-treat analyses with low attrition and were rated low risk. Outcome measurement was rated low risk in all studies: primary efficacy endpoints (e.g., PFS, OS) were evaluated by a blinded independent review committee (IRRC/BICR) or were inherently objective. Risk of selective reporting was also deemed low across trials; all were prospectively registered and reported prespecified primary and secondary outcomes in the published manuscripts. Detailed, domain-level assessments are presented in **Figure 2**.

### 3.3 Head-to-head meta-analysis

#### 3.3.1 Comparisons of grade ≥3 trAEs, any irAEs and grade ≥3 irAEs

Seven trials reported grade ≥3 trAEs. Between-study heterogeneity was modest (*P*< 0.10; *I²* = 50%), so a random-effect model was applied. For patients with advanced ESCC, ICIs combined with chemotherapy were associated with a higher incidence of grade ≥3 trAEs compared with chemotherapy alone (RR = 1.08; 95% CI, 1.00-1.17).Six trials reported any-grade irAEs. Heterogeneity was substantial (*P*< 0.10; *I²* = 71%); therefore, a random-effects model was used. Immunochemotherapy significantly increased the risk of any-grade irAEs (RR = 2.04, 95% CI 1.71-2.44).For grade ≥3 irAEs, no between-study heterogeneity was detected (*P*> 0.10; *I²* = 2%), and a fixed-effect model was adopted. Immunochemotherapy significantly increased the risk of grade ≥3 irAEs (RR = 2.75, 95% CI 1.98-3.82). Detailed results are shown in **Figure 2** and **Supplementary Figures S1**-**Supplementary Figures S3**.

#### 3.3.2 Comparison of immune-mediated adverse events: rash, thyroid dysfunction, and pneumonitis

Six studies reported immune-mediated rash; no between-study heterogeneity was detected (*P*> 0.10; *I²* = 0%), so a fixed-effect model was applied. Compared with chemotherapy alone, immunochemotherapy increased the risk of immune-mediated rash (RR = 3.68, 95% CI 2.55-5.32).

Six studies reported immune-mediated hypothyroidism; heterogeneity was absent (*P*> 0.10; *I²* = 0%), and a fixed-effect model was used. Immunochemotherapy was associated with a higher risk of immune-mediated hypothyroidism (RR = 2.35, 95% CI 1.76-3.14).

Five studies reported immune-mediated hyperthyroidism; heterogeneity was likewise absent (*P* > 0.10; *I²* = 0%), warranting a fixed-effect model. Immunochemotherapy increased the risk of immune-mediated hyperthyroidism (RR = 3.35, 95% CI 1.93-5.82).

Five studies reported immune-mediated pneumonitis; heterogeneity was modest (*P*> 0.10; *I²* = 0%), and a fixed-effect model was applied. Compared with chemotherapy alone, ICIs combined with chemotherapy appeared to increase the risk of immune-mediated pneumonitis in patients with advanced ESCC; however, the difference did not reach statistical significance (RR = 1.41; 95% CI, 0.84-2.37).

**Figure 3:**
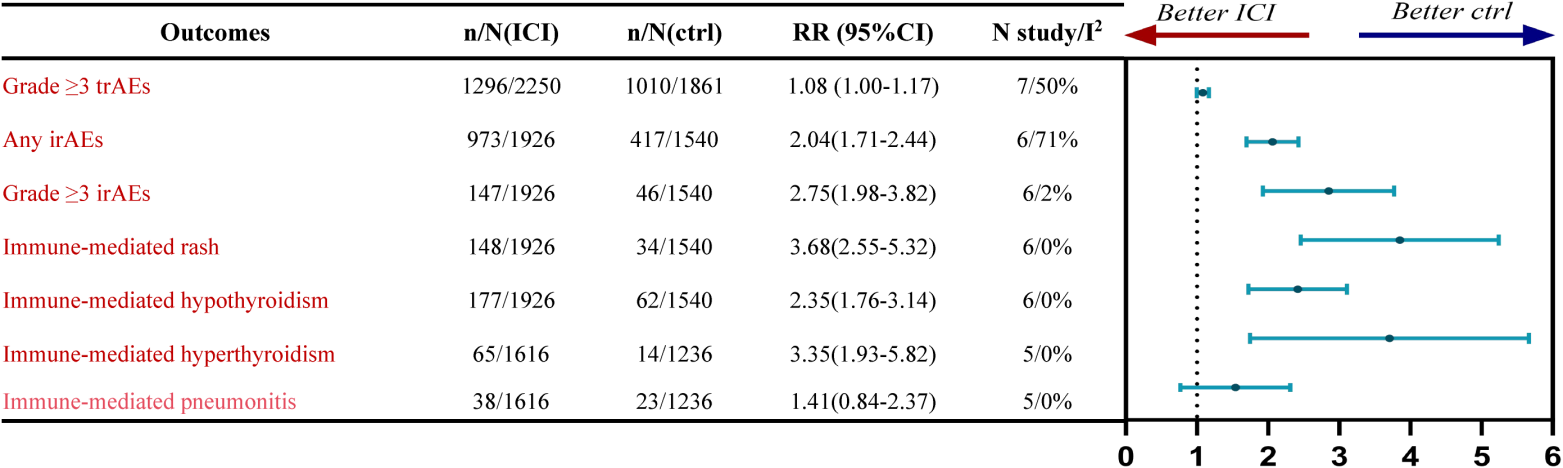
Forest plot showing the comparative risk of severe and immune-related adverse events between first-line ICI therapy and chemotherapy in advanced ESCC.

These findings are summarized in **Figure 3**, with additional details provided in **Supplementary Figures S4**-**Supplementary Figures S7**.

#### 3.3.3 Certainty of evidence (GRADE)

The certainty of evidence for safety outcomes was assessed according to the GRADE framework (**Table 2**). For grade ≥3 trAEs, the certainty was rated as moderate, primarily because of moderate between-study heterogeneity and borderline imprecision, as the confidence interval approached the null effect. The certainty for any-grade immune-related adverse events was likewise downgraded to moderate due to substantial heterogeneity across trials. In contrast, the evidence for Grade ≥3 immune-related adverse events, immune-mediated rash, hypothyroidism, and hyperthyroidism was rated as high certainty, supported by consistent results across studies, relatively large effect sizes, and narrow confidence intervals. For immune-mediated pneumonitis, the certainty of evidence was judged to be low. This rating reflected imprecision arising from a limited number of events and wide confidence intervals that encompassed the possibility of no effect, leading to considerable uncertainty regarding the true magnitude and direction of the association.

**Table 2:** SoF table for safety endpoints.

Outcome	Control risk (per 1000)	Corresponding risk (per 1000)	Absolute effect (95% CI, per 1000)	Relative effect (RR, 95% CI)	n (studies)	Certainty (GRADE)
Grade ≥3 trAEs	543	586 (543-635)	43 more (0 to 92 more)	1.08 (1.00-1.17)	4111(7 RCTs)	⊕⊕⊕⊖Moderate^a^
Any-grade irAEs	271	552 (463-661)	282 more (192 to 390 more)	2.04 (1.71-2.44)	3466(6 RCTs)	⊕⊕⊕⊖Moderate^a^
Grade ≥3 irAEs	30	82 (59-114)	52 more (29 to 84 more)	2.75 (1.98-3.82)	3466(6 RCTs)	⊕⊕⊕⊕high
Immune-mediated rash	22	81 (56-117)	59 more (34 to 95 more)	3.68 (2.55-5.32)	3466(6 RCTs)	⊕⊕⊕⊕high
Immune-mediated hypothyroidism	40	95 (71-126)	54 more (31 to 86 more)	2.35 (1.76-3.14)	3466(6 RCTs)	⊕⊕⊕⊕high
Immune-mediated hyperthyroidism	11	38 (22-66)	27 more (11 to 55 more)	3.35 (1.93-5.82)	2852(5 RCTs)	⊕⊕⊕⊕high
Immune-mediated pneumonitis	19	26 (16-44)	8 more (3 fewer to 25 more)	1.41 (0.84-2.37)	2852(5 RCTs)	⊕⊕⊖⊖Low^b^

All risks are per 1,000; values are point estimate (95% CI).

a: Imprecision — 95% CI crosses the pre-specified absolute MID.

b: Imprecision — few events and wide confidence intervals crossing the null, resulting in uncertain effect estimates.

### 3.4 Network meta-analyses

#### 3.4.1 Comparisons of grade ≥3 trAEs, any irAEs and grade ≥3 irAEs

**Figure 4:**
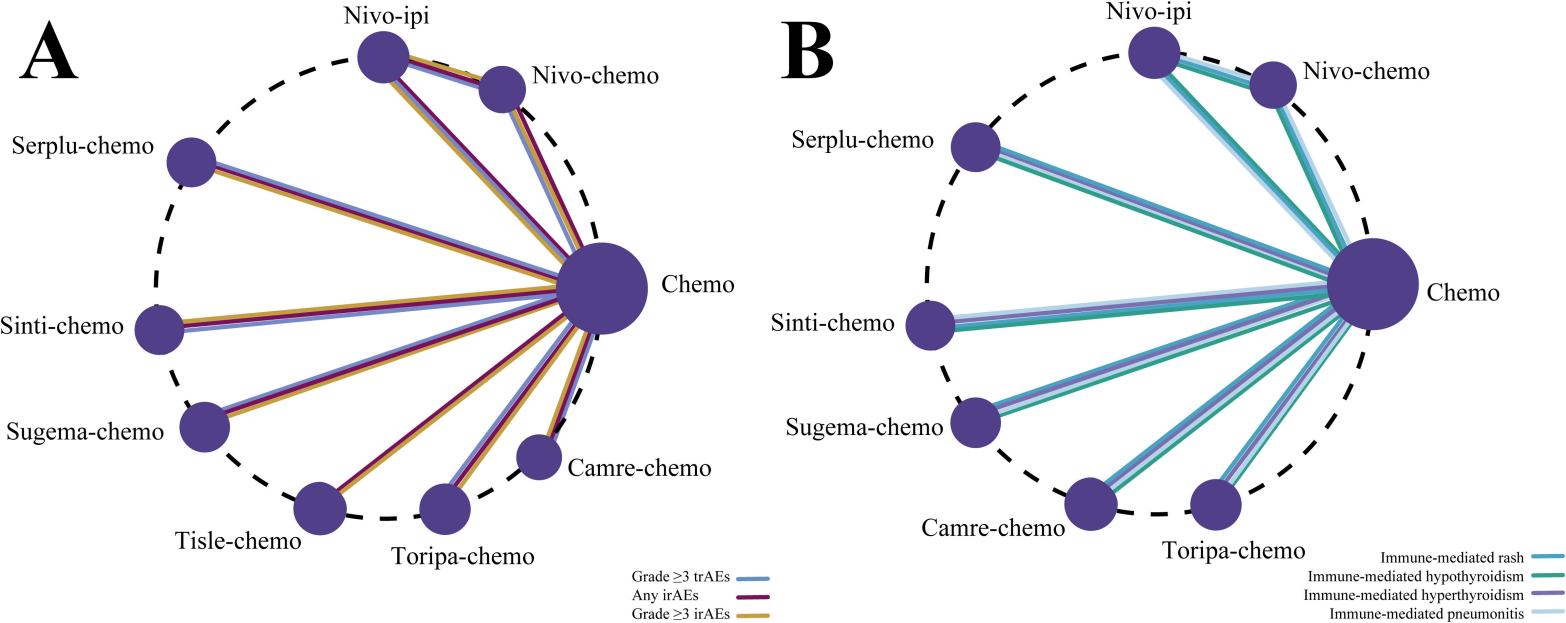
Network diagrams of comparative safety profiles for first-line immunotherapy regimens in advanced ESCC. **(A)** Network plot comparing treatment regimens based on grade ≥3 trAEs, any irAEs, and Grade ≥3 irAEs. **(B)** Network plot comparing regimens for specific irAE subtypes, including rash, hypothyroidism, hyperthyroidism, and pneumonitis.

The primary outcomes of this NMA included the incidence of grade ≥3 trAEs, any-grade irAEs, and grade ≥3 irAEs among patients with advanced ESCC. Analyses of grade ≥3 trAEs included eight ICI-based regimens, whereas analyses of any-grade and Grade ≥3 immune-related adverse events involved six regimens (**Figure 4A**).

**Figure 5:**
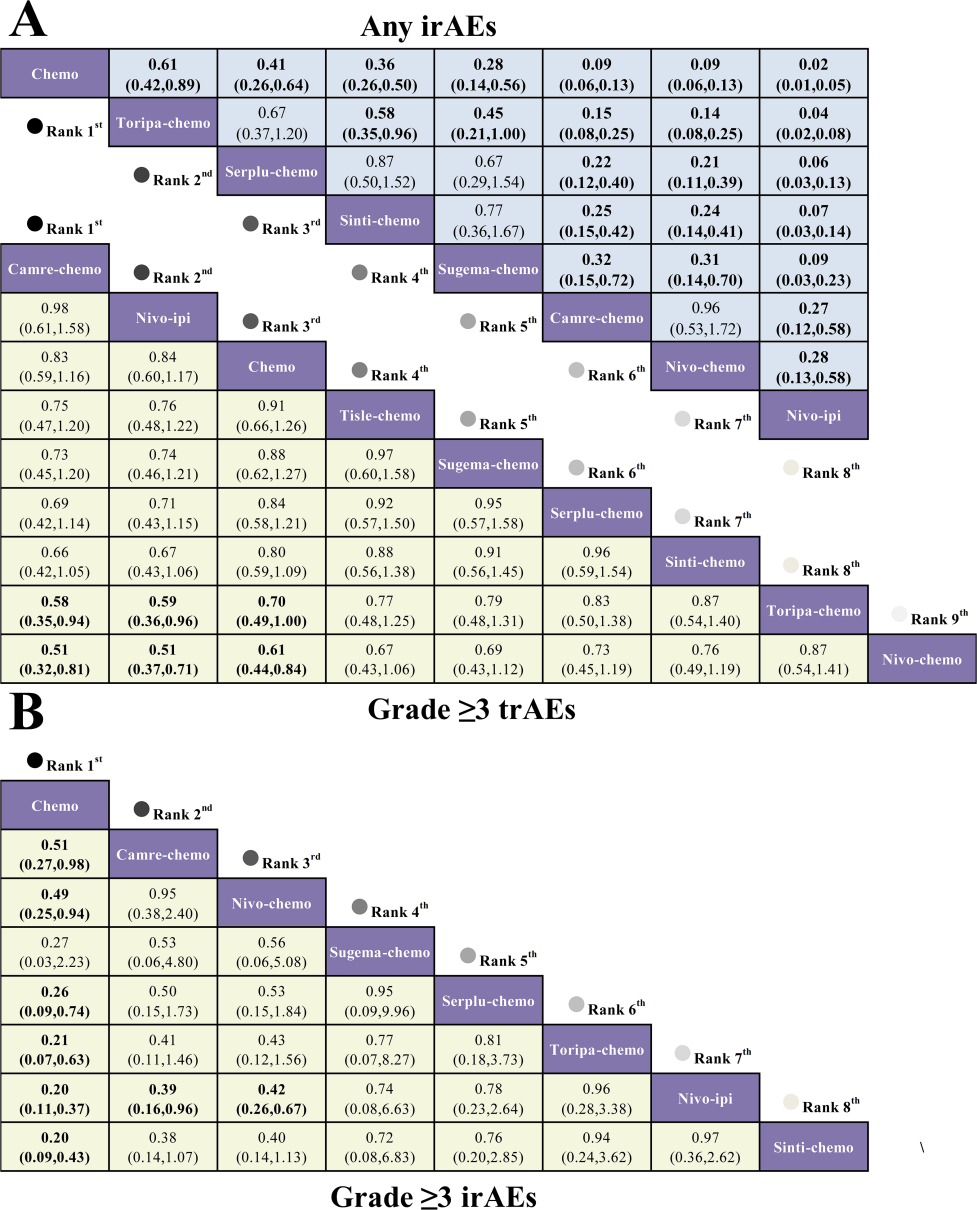
League tables of serious and irAEs for first-line immunotherapy regimens in advanced ESCC. **(A)** League table comparing the RR and 95% CIs of any irAEs and grade ≥ 3 trAEs between immunotherapy regimens. **(B)** League table of grade ≥ 3 irAEs. RR < 1.00 indicates a lower risk (better safety profile) for the treatment in the row compared to the treatment in the column.

For grade ≥3 trAEs, only camre-chemo (RR = 0.83, 95% CI 0.59-1.16) and nivo-ipi (RR = 0.84, 95% CI 0.60-1.17) showed numerically lower rates than chemotherapy, although neither comparison reached statistical significance. For any irAEs, all first-line ICI-based regimens were associated with a significantly higher risk compared with chemotherapy. Relative to nivo-ipi, both chemotherapy (RR = 0.02, 95% CI 0.01-0.05) and toripa-chemo (RR = 0.04, 95% CI 0.02-0.08) markedly reduced the incidence of any-grade irAEs, whereas the remaining pairwise comparisons are presented in **Figure 5A**. For grade ≥3 irAEs, all first-line ICI-based regimens significantly increased the risk compared with chemotherapy. When sinti-chemo was used as the comparator, chemotherapy showed the lowest risk (RR = 0.20, 95% CI 0.09-0.43). The risk of grade ≥3 irAEs was similar between sinti-chemo and nivo-ipi (RR = 0.97, 95% CI 0.36-2.62), as illustrated in **Figure 5B**.

#### 3.4.2 Comparative analysis of immune-related adverse events: rash, thyroid dysfunction, and pneumonitis

The secondary outcomes of this NMA focused on specific irAEs, including rash, hypothyroidism, hyperthyroidism, and pneumonitis. Data from patients with advanced ESCC were pooled across studies reporting on 7, 7, 7, and 5 ICI-based regimens, respectively, for each outcome (**Figure 4B**).

**Figure 6:**
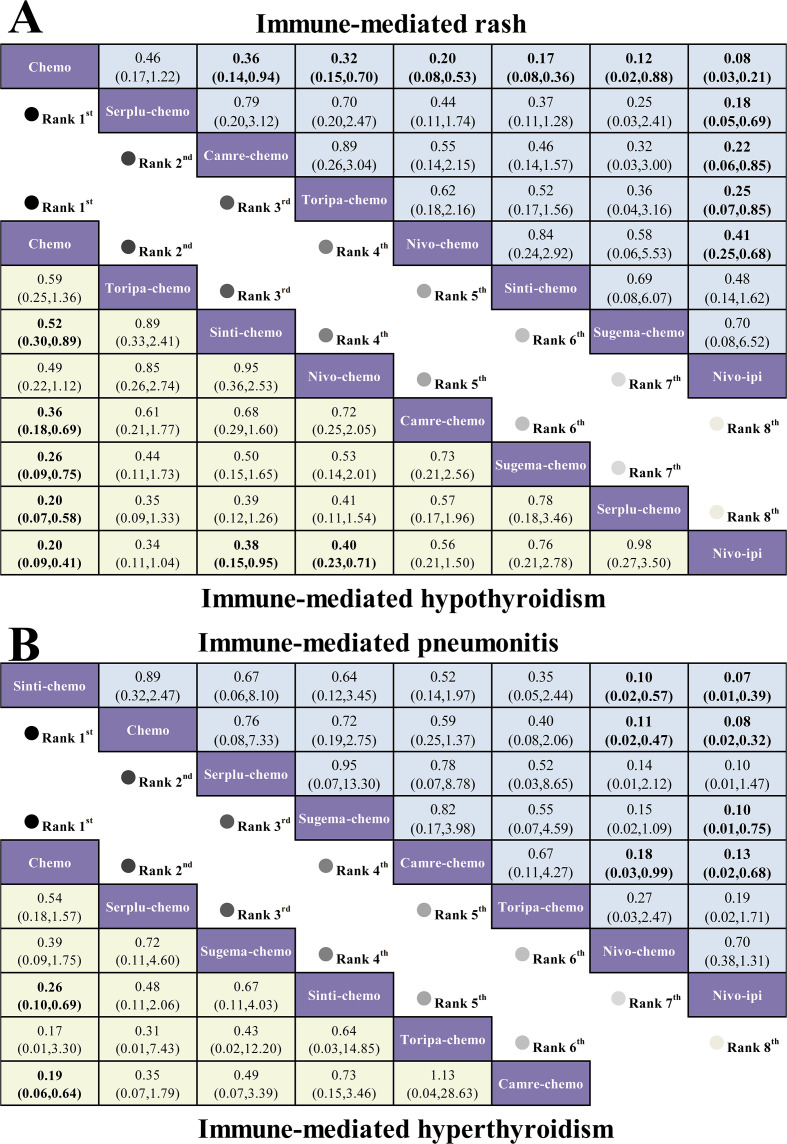
League tables of typical irAEs for first-line immunotherapy regimens in advanced ESCC. **(A)** League table comparing the RR and 95% CI of immune-mediated rash and hypothyroidism between different first-line immunotherapy regimens. **(B)** League table comparing immune-mediated hyperthyroidism and pneumonitis.

For immune-mediated rash, chemotherapy demonstrated the lowest incidence compared with nivo-ipi (RR = 0.08, 95% CI 0.03-0.21). Serplu-chemo (RR = 0.18, 95% CI 0.05-0.69) and camre-chemo (RR = 0.22, 95% CI 0.06-0.85) ranked next, each showing a significant reduction in risk.For immune-mediated hypothyroidism, chemotherapy again had the lowest incidence relative to nivo-ipi (RR = 0.20, 95% CI 0.09-0.41). Sinti-chemo (RR = 0.38, 95% CI 0.15-0.95) and nivo-chemo (RR = 0.40, 95% CI 0.23-0.71) also demonstrated lower risks, whereas the remaining comparisons are presented in **Figure 6A**.

For immune-mediated hyperthyroidism, chemotherapy showed the lowest incidence when compared with camre-chemo (RR = 0.19, 95% CI 0.06-0.64). Toripa-chemo also demonstrated a numerically lower risk (RR = 0.17, 95% CI 0.01-3.30), although this difference was not statistically significant. For immune-mediated pneumonitis, sinti-chemo had the lowest incidence relative to nivo-ipi (RR = 0.07, 95% CI 0.01 to 0.39), followed closely by chemotherapy (RR = 0.08, 95% CI 0.02-0.32), as shown in **Figure 6B**.

### 3.5 SUCRA ranking of safety profiles among first line regimens

According to SUCRA-based ranking of safety profiles, camre-chemo had the highest probability of being the regimen with the lowest risk of Grade ≥3 trAEs in patients with advanced ESCC (SUCRA = 87.8%), followed by nivo-ipi (86.4%) and Themotherapy alone (68.7%). For any irAEs, excluding chemotherapy, toripa-chemo had the greatest probability of yielding the lowest risk (83.8%), followed by serplu-chemo (65.5%). With respect to grade ≥3 irAEs, again excluding chemotherapy, camre-chemo ranked as the safest regimen (71.6%), followed by nivo-chemo.

**Figure 7:**
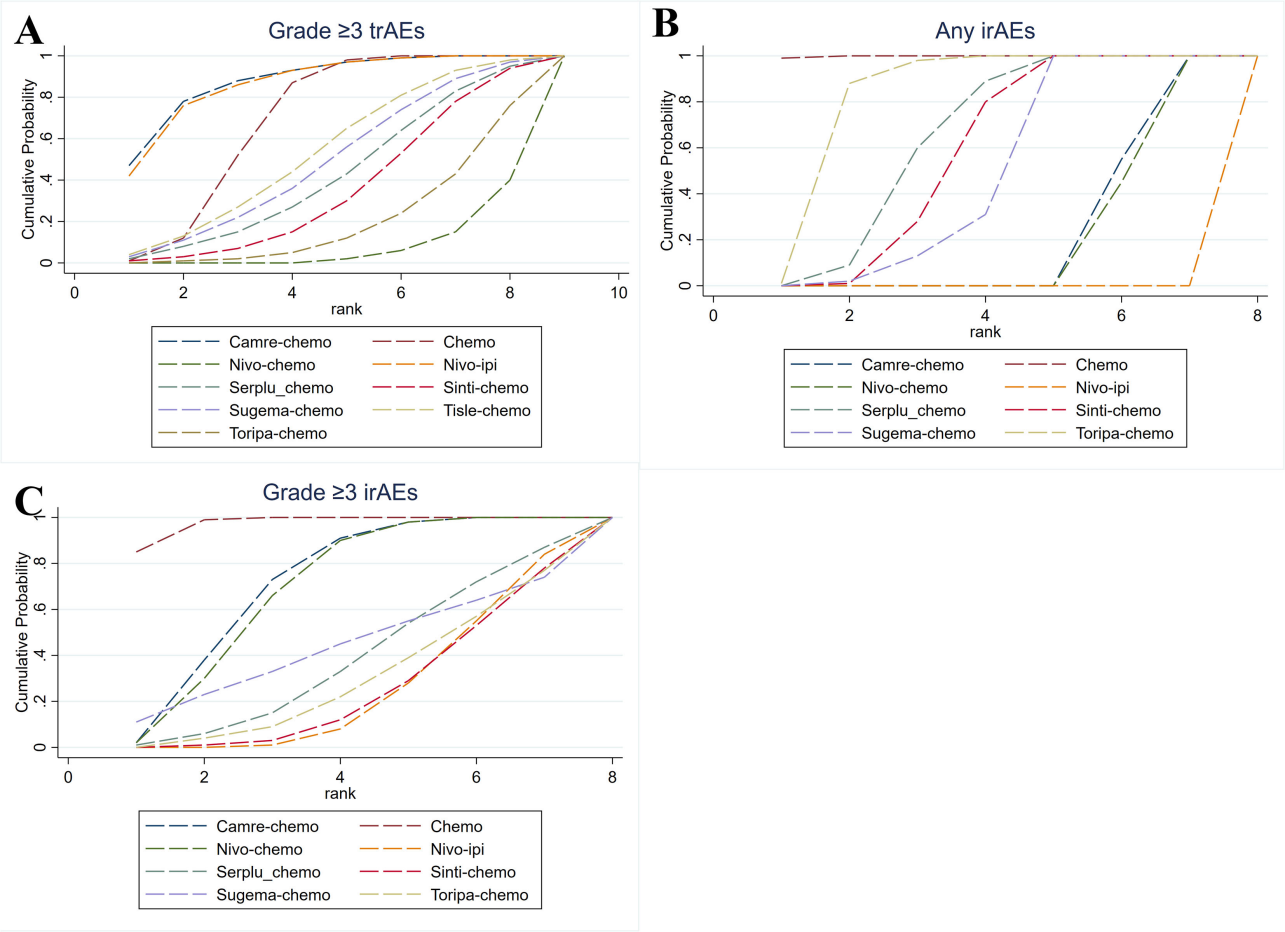
Bayesian cumulative ranking curves for serious and irAEs of first-line immunotherapy in advanced ESCC. **(A)** Cumulative ranking curves for grade ≥3 trAEs. **(B)** Cumulative ranking curves for any irAEs. **(C)** Cumulative ranking curves for grade ≥3 irAEs.

**Figure 8:**
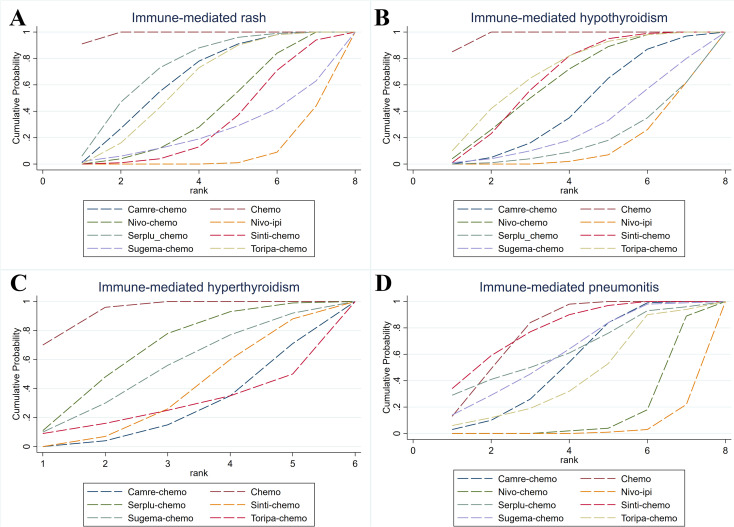
Bayesian cumulative ranking curves for typical irAEs of first-line immunotherapy in advanced ESCC. **(A)** Cumulative ranking curves for immune-mediated rash. **(B)** Cumulative ranking curves for immune-mediated hypothyroidism. **(C)** Cumulative ranking curves for immune-mediated hyperthyroidism. **(D)** Cumulative ranking curves for immune-mediated pneumonitis.

We further evaluated common immune-mediated adverse events. For immune-mediated rash, apart from chemotherapy, serplu-chemo showed the lowest risk (72.7%). For immune-mediated hypothyroidism, toripa-chemo (69.8%) and sinti-chemo (65.0%) had the most favorable safety rankings. For immune-mediated hyperthyroidism, excluding chemotherapy, serplu-chemo ranked lowest in risk (65.5%), followed by sugema-chemo (52.5%). For immune-mediated pneumonitis, sinti-chemo had the highest probability of being the safest regimen (79.6%), followed by chemotherapy (77.6%) and serplu-chemo (63.6%). Detailed ranking information can be found in **Figures 7**, **8**, **Supplementary Table S3**.

### 3.6 Assessment of heterogeneity, inconsistency, sensitivity, and publication bias

Model convergence was assessed using diagnostic trace plots and the Gelman-Rubin statistic. The results indicated stable and reproducible MCMC simulations across all outcomes, supporting reliable convergence of the Bayesian models (**Supplementary Figures 15**-**28**). To evaluate model fit and the robustness of the findings, both fixed-effects and random-effects network meta-analysis models were constructed and compared using the deviance information criterion (DIC), the posterior mean residual deviance (D̄), and the effective number of parameters (pD). As shown in **Supplementary Table S4**, the DIC differences between fixed-effects and random-effects models were consistently below 5 across all endpoints (e.g., grade ≥3 trAEs: 29.91 vs. 29.91; any irAEs: 25.83 vs. 25.85), indicating comparable goodness-of-fit and supporting the stability of the model estimates.

Network consistency was further examined by comparing the DIC values of consistency and inconsistency models. As presented in **Supplementary Table S5**, all endpoints exhibited DIC differences of less than 5 (e.g., grade ≥3 trAEs: 30.06 vs. 29.85; any irAEs: 26.06 vs. 25.80), suggesting no meaningful inconsistency between direct and indirect evidence and supporting the plausibility of the transitivity assumption across the network. In addition, between-study heterogeneity was low, with I² values ranging from 7% to 20%, indicating a high degree of homogeneity and a structurally coherent network.

In the pairwise meta-analyses, heterogeneity was evaluated using Cochran’s Q test and the I² statistic. We also conducted leave-one-out sensitivity analyses by sequentially excluding individual studies. These analyses did not materially change the heterogeneity estimates or the pooled effect sizes, which supports the robustness of the summary results.Potential publication bias was assessed visually using funnel plots for the main outcomes (grade ≥3 trAEs, any-grade irAEs, and grade ≥3 irAEs). The funnel plots appeared largely symmetrical without marked asymmetry or extreme outliers, suggesting a low likelihood of substantial publication bias (**Supplementary Figures 29**-**35**).

Finally, we performed a univariable network meta-regression with chemotherapy regimen as a covariate to explore whether different chemotherapy backbones modified the relative effects of immunotherapy-containing regimens versus chemotherapy alone. No statistically significant associations were observed between chemotherapy regimen and the treatment effect of any intervention relative to chemotherapy (**Supplementary Table S7**).

## 4 Discussion

### 4.1 Major findings

To our knowledge, this is the first study to prioritize regimen-level risk ranking for grade ≥3 trAEs and immune-related toxicities in advanced ESCC. It offers decision-oriented evidence for practice, specifically:

1. In patients with advanced ESCC, ICI-based chemotherapy was associated with a significantly higher risk of grade ≥3 treatment-related adverse events, any-grade irAEs, and grade ≥3 irAEs compared with chemotherapy alone.
2. ICI-based regimens also significantly increased the incidence of immune-mediated rash, hypothyroidism, and hyperthyroidism. For immune-mediated pneumonitis, the risk was numerically higher but did not reach statistical significance.
3. Among all ICI-chemotherapy combinations, camre-chemo demonstrated the most favorable safety profile, with the lowest probabilities of grade ≥3 trAEs and Grade ≥3 irAEs, whereas toripa-chemo was associated with the lowest probability of any-grade irAEs.
4. Nivo-ipi required particular vigilance given its high irAE burden, especially immune-mediated rash, hypothyroidism, and pneumonitis. Among ICI-chemotherapy regimens, nivo-chemo warranted heightened monitoring for immune-mediated pneumonitis and rash, while sinti-chemo required close attention to thyroid-related toxicities.

Compared with chemotherapy alone, ICI-based chemotherapy regimens were associated with a significantly increased overall risk of Grade ≥3 trAEs (RR = 1.08) and irAEs (RR = 2.04). This elevation in risk is biologically plausible, as ICIs release inhibitory checkpoints on T cells and thereby activate broad antigen-specific and non-specific T cell responses. While this augments antitumor immunity and enhances tumor antigen recognition, it also disrupts peripheral immune tolerance and can trigger autoimmune-like reactions, leading to a marked increase in the risk of irAEs (14).Cytotoxic agents (e.g., taxanes, platinum compounds) not only exert direct tumoricidal effects but also induce immunogenic cell death (ICD), promoting the release of tumor-associated antigens and “danger signals” (e.g., HMGB1, ATP, calreticulin), thereby augmenting antigen presentation and T-cell priming (25).In combination with ICIs, these mechanisms synergistically amplify antitumor immunity but can also propagate systemic immune activation and off-target inflammation in healthy tissues (26). This mechanism is further influenced by the tumor microenvironment, which modulates immune toxicity. Emerging evidence suggests that the tumor microenvironment, including immune and stromal compartments, significantly shapes the immune system’s response to cancer therapies. Tumor cells can influence immune function through metabolic reprogramming, a process mediated by extracellular vesicles that play a pivotal role in immune modulation (27). This perspective underscores that irAEs should not only be viewed as off-target effects but as reflections of systemic immune rewiring, influenced by tumor-derived signals and immune system interactions.

Additionally, this study may help inform the development of next-generation treatment regimens and the identification of biomarkers by clarifying how differential toxicity profiles shape therapeutic strategies (28). By delineating regimen-specific safety patterns, our findings can support more nuanced, toxicity-aware decision-making and the design of future trials that seek to optimise both efficacy and tolerability. The SUCRA-based toxicity rankings quantify the relative risk of different regimens and may help clinicians understand comparative safety profiles, which should be weighed alongside regimen-specific efficacy data, regulatory labels, PD-L1 expression status, cost, and patient preferences when selecting first-line therapy.

For example, nivo-ipi was ranked highest with respect to avoiding grade ≥3 trAEs (SUCRA = 86.4) but carried a higher probability of immune-related adverse events, particularly rash and hypothyroidism, and therefore requires close monitoring of immune-mediated toxicities. In contrast, camre-chemo exhibited a more balanced profile, with relatively favourable rankings for both grade ≥3 trAEs and grade ≥3 irAEs. These patterns suggest that camrelizumab plus chemotherapy may represent a safer option among immunochemotherapy regimens from a toxicity standpoint, whereas nivolumab-ipilimumab may be considered in clinical scenarios where avoidance of chemotherapy-related systemic toxicities is prioritised, provided that the increased risk of irAEs is carefully managed. Importantly, these interpretations are based solely on safety rankings and are not intended as stand-alone treatment recommendations.

The higher risk of grade ≥3 irAEs with immunochemotherapy (RR = 2.75) underscores the inherent tension between therapeutic immune activation and maintenance of immune homeostasis, with attendant organ-specific injury. Dual checkpoint blockade with nivo-ipi avoids chemotherapy-related systemic toxicities (e.g., myelosuppression, gastrointestinal reactions, hepatic and renal injury), which may underlie its lower probability of grade ≥3 trAEs; however, concomitant PD-1 and CTLA-4 inhibition intensifies effector T-cell activation and impairs Treg function, further eroding self-tolerance and increasing the frequency and severity of irAEs such as rash and hypothyroidism (29). This dual mechanism of immune modulation further highlights the evolving approach to cancer therapy, in which the goal is not merely to eliminate cancer cells but to fine-tune immune responses. The future of cancer immunotherapy may lie in strategies that modulate immune reactivity to balance tumour control with minimal collateral tissue damage (30, 31). The direct associations between immune activation and organ-specific toxicities such as thyroid dysfunction (e.g., hypothyroidism), dermatologic effects (e.g., rash), and pneumonitis reflect the close interplay between immune system modulation and susceptibility of target organs.

From a benefit-risk perspective, it is also important to recognise that the regimens included in this network do not confer identical OS, PFS, or ORR benefits, and that their regulatory indications and reimbursement status differ across regions and PD-L1 subgroups. Our analysis was restricted to safety endpoints and does not incorporate comparative efficacy; therefore, the SUCRA-based toxicity rankings should be interpreted as complementary information to existing efficacy data and clinical guidelines rather than as prescriptive recommendations. In practice, regimen selection for first-line advanced ESCC should be guided primarily by high-quality evidence on survival benefit, guideline recommendations, PD-L1 status, comorbidities, and patient preferences, with our safety-focused rankings serving as one component of an integrated benefit-risk assessment.

Consistent with the meta-analysis by Ren et al., our results support a higher risk of grade ≥3 trAEs with first-line immunochemotherapy relative to chemotherapy, while extending prior work by delineating regimen-specific irAE spectra in greater detail (32).

### 4.2 Implications

Methodologically, this Bayesian network meta-analysis is among the most comprehensive safety syntheses to date for first-line immunotherapy in advanced ESCC, encompassing seven high-quality RCTs, 4,479 patients, and nine competing strategies. Beyond aggregate endpoints (grade ≥3 trAEs/irAEs), we characterized organ-specific risks (rash, thyroid dysfunction, immune-mediated pneumonitis), addressing a key gap in safety evaluation. Adherence to PRISMA-NMA guidance, Bayesian modeling, and SUCRA-based ranking strengthens the robustness and interpretability of indirect comparisons across regimens. Notably, the dual profile of nivo-ipi—lower probability of grade ≥3 trAEs but higher irAE liability—illustrates how regimen-level trade-offs can inform individualized treatment selection and proactive toxicity management.

### 4.3 Limitations

This study has limitations. First, despite comprehensive literature searches, the number of eligible RCTs was limited, which may constrain the precision of some estimates; nonetheless, the overall sample size was substantial and the included trials were generally well conducted. Second, the current network is essentially star-shaped, with most ICI-based regimens compared only against chemotherapy rather than directly against each other. Consequently, the relative rankings and indirect comparisons rely heavily on the transitivity assumption rather than head-to-head evidence, and should be interpreted with appropriate caution. However, enrolled populations were broadly similar across trials with respect to line of therapy (all first-line), ECOG performance status, disease stage, and platinum-based chemotherapy backbones, which supports the clinical plausibility of these indirect comparisons. Third, the geographic representation of the included trials limits the generalizability of our findings. As summarised in **Table 1**, several key studies were conducted predominantly in China or the broader Asia-Pacific region, whereas others were global but still enrolled few patients from Africa or the so-called ‘esophageal cancer belt’ outside East Asia. Given that the burden of ESCC is particularly high in East Asia and along this belt, our safety conclusions are largely driven by cohorts with a high proportion of Chinese and Asia-Pacific patients and may not fully capture toxicity profiles in under-represented regions. Moreover, all data were derived from RCT settings with relatively preserved organ function, strict eligibility criteria, and fewer comorbidities than typically seen in routine practice. Older patients and those with multiple comorbid conditions, poorer performance status, or limited access to supportive care may experience a different spectrum and severity of irAEs and chemotherapy-related toxicities, which could affect the external validity of our results. Additionally, another limitation arises from the varying definitions of AEs across trials, as well as the underreporting of late-onset irAEs, both of which could impact the accuracy and generalizability of the findings. Moreover, it is important to note that SUCRA-based rankings depend on the network structure and should not be interpreted as an absolute safety hierarchy. These rankings represent relative comparisons within the network and may vary with the inclusion of additional data or different network configurations. Finally, open-label designs in studies such as ESCORT-1st and CheckMate 648 introduce the potential for bias, as knowledge of treatment allocation could have influenced co-interventions or other behaviors. This concern is reflected in the RoB2 assessment, particularly in terms of deviations from intended interventions.

## Data Availability

The original contributions presented in the study are included in the article/**Supplementary Material**. Further inquiries can be directed to the corresponding author.

## References

[1] Bray F Laversanne M Sung H Ferlay J Siegel RL Soerjomataram I . Global cancer statistics 2022: GLOBOCAN estimates of incidence and mortality worldwide for 36 cancers in 185 countries. CA: Cancer J Clin. (2024) 74:229–63. doi: 10.3322/caac.21834, PMID: 38572751

[2] Abnet CC Arnold M Wei W-Q . Epidemiology of esophageal squamous cell carcinoma. Gastroenterology. (2018) 154:360–73. doi: 10.1053/j.gastro.2017.08.023, PMID: 28823862 PMC5836473

[3] Deboever N Jones CM Yamashita K Ajani JA Hofstetter WL . Advances in diagnosis and management of cancer of the esophagus. BMJ 385. (2024). 385:e074962doi: 10.1136/bmj-2023-074962, PMID: 38830686

[4] Ohashi S Miyamoto SI Kikuchi O Goto T Amanuma Y Muto XXXM . Recent advances from basic and clinical studies of esophageal squamous cell carcinoma. Gastroenterology. (2015) 149:1700–15. doi: 10.1053/j.gastro.2015.08.054, PMID: 26376349

[5] Vellayappan BA Soon YY Ku GY Leong CN Lu JJ Tey JC . Chemoradiotherapy versus chemoradiotherapy plus surgery for esophageal cancer. Cochrane Database Systematic Rev. (2017). 8:CD010511. doi: 10.1002/14651858.CD010511.pub2, PMID: 28829911 PMC6483706

[6] Sonkin D Thomas A Teicher BA . Cancer treatments: Past, present, and future. Cancer Genet. (2024) 286:18–24. doi: 10.1016/j.cancergen.2024.06.002, PMID: 38909530 PMC11338712

[7] Shoji Y Koyanagi K Kanamori K Tajima K Ogimi M Ninomiya Y . Immunotherapy for esophageal cancer: where are we now and where can we go. World J Gastroenterol. (2024) 30:2496. doi: 10.3748/wjg.v30.i19.2496, PMID: 38817664 PMC11135418

[8] Ribas A Wolchok JD . Cancer immunotherapy using checkpoint blockade. Science. (2018) 359:1350–5. doi: 10.1126/science.aar4060, PMID: 29567705 PMC7391259

[9] Kumar V Chaudhary N Garg M Floudas CS Soni P Chandra AB . Current diagnosis and management of immune related adverse events (irAEs) induced by immune checkpoint inhibitor therapy. Front Pharmacol. (2017) 8:49. doi: 10.3389/fphar.2017.00049, PMID: 28228726 PMC5296331

[10] Darnell EP Mooradian MJ Baruch EN Yilmaz M Reynolds KL . Immune-related adverse events (irAEs): diagnosis, management, and clinical pearls. Curr Oncol Rep. (2020) 22:39. doi: 10.1007/s11912-020-0897-9, PMID: 32200442

[11] Sun J-M Shen L Shah MA Enzinger P Adenis A Doi T . Pembrolizumab plus chemotherapy versus chemotherapy alone for first-line treatment of advanced oesophageal cancer (KEYNOTE-590): a randomised, placebo-controlled, phase 3 study. Lancet. (2021) 398:759–71. doi: 10.1016/S0140-6736(21)01234-4, PMID: 34454674

[12] Luo H Lu J Bai Y Mao T Wang J Fan Q . Final analysis of the randomized phase 3 ESCORT-1st trial: Camrelizumab plus chemotherapy as first-line treatment for advanced or metastatic esophageal squamous cell carcinoma. Am Soc Clin Oncol. (2024). 42:4055–4055. doi: 10.1200/JCO.2024.42.16_suppl.4055

[13] Lu Z Wang J Shu Y Liu L Kong L Yang L . Sintilimab versus placebo in combination with chemotherapy as first line treatment for locally advanced or metastatic oesophageal squamous cell carcinoma (ORIENT-15): multicentre, randomised, double blind, phase 3 trial. BMJ 377. (2022). 377:e068714. doi: 10.1136/bmj-2021-068714, PMID: 35440464 PMC9016493

[14] Postow MA Sidlow R Hellmann MD . Immune-related adverse events associated with immune checkpoint blockade. New Engl J Med. (2018) 378:158–68. doi: 10.1056/NEJMra1703481, PMID: 29320654

[15] Martins F Sofiya L Sykiotis GP Lamine F Maillard M Fraga M . Adverse effects of immune-checkpoint inhibitors: epidemiology, management and surveillance. Nat Rev Clin Oncol. (2019) 16:563–80. doi: 10.1038/s41571-019-0218-0, PMID: 31092901

[16] Haanen J Carbonnel F Robert C Kerr K Peters S Larkin J . Management of toxicities from immunotherapy: ESMO Clinical Practice Guidelines for diagnosis, treatment and follow-up. Ann Oncol. (2017) 28:iv119–42. doi: 10.1093/annonc/mdx225, PMID: 28881921

[17] Hutton B Catala-Lopez F Moher D . The PRISMA statement extension for systematic reviews incorporating network meta-analysis: PRISMA-NMA. Med Clínica (English Edition). (2016) 147:262–6. doi: 10.1016/j.medcle.2016.10.003 27040178

[18] Sadeghirad B Foroutan F Zoratti MJ Busse JW Brignardello-Petersen R Guyatt G . Theory and practice of Bayesian and frequentist frameworks for network meta-analysis. BMJ Evidence-Based Med. (2023) 28:204–9. doi: 10.1136/bmjebm-2022-111928, PMID: 35760451

[19] Sterne JA Savović J Page MJ Elbers RG Blencowe NS Boutron I . RoB 2: a revised tool for assessing risk of bias in randomised trials. bmj. (2019) 366:l4898. doi: 10.1136/bmj.l4898, PMID: 31462531

[20] Wang Z-X Cui C Yao J Zhang Y Li M Feng J . Toripalimab plus chemotherapy in treatment-naïve, advanced esophageal squamous cell carcinoma (JUPITER-06): A multi-center phase 3 trial. Cancer Cell. (2022) 40:277–288. e3. doi: 10.1016/j.ccell.2022.02.007, PMID: 35245446

[21] Doki Y Ajani JA Kato K Xu J Wyrwicz L Motoyama S . Nivolumab combination therapy in advanced esophageal squamous-cell carcinoma. New Engl J Med. (2022) 386:449–62. doi: 10.1056/NEJMoa2111380, PMID: 35108470

[22] Song Y Zhang B Xin D Kou X Tan Z Zhang S . First-line serplulimab or placebo plus chemotherapy in PD-L1-positive esophageal squamous cell carcinoma: a randomized, double-blind phase 3 trial. Nat Med. (2023) 29:473–82. doi: 10.1038/s41591-022-02179-2, PMID: 36732627 PMC9941045

[23] Li J Chen Z Bai Y Liu B Li Q Zhang J . First-line sugemalimab with chemotherapy for advanced esophageal squamous cell carcinoma: a randomized phase 3 study. Nat Med. (2024) 30:740–8. doi: 10.1038/s41591-024-02797-y, PMID: 38302715

[24] Xu J Kato K Raymond E Hubner RA Shu Y Pan Y . Tislelizumab plus chemotherapy versus placebo plus chemotherapy as first-line treatment for advanced or metastatic oesophageal squamous cell carcinoma (RATIONALE-306): a global, randomised, placebo-controlled, phase 3 study. Lancet Oncol. (2023) 24:483–95. doi: 10.1016/S1470-2045(23)00108-0, PMID: 37080222

[25] Derosa L Routy B Fidelle M Iebba V Alla L Pasolli E . Gut bacteria composition drives primary resistance to cancer immunotherapy in renal cell carcinoma patients. Eur Urol. (2020) 78:195–206. doi: 10.1016/j.eururo.2020.04.044, PMID: 32376136

[26] Kepp O Tesniere A Schlemmer F Michaud M Senovilla L Zitvogel L . Immunogenic cell death modalities and their impact on cancer treatment. Apoptosis. (2009) 14:364–75. doi: 10.1007/s10495-008-0303-9, PMID: 19145485

[27] Encarnação CC Faria GM Franco VA Botelho LGX Moraes JA Renovato-Martins M . Interconnections within the tumor microenvironment: extracellular vesicles as critical players of metabolic reprogramming in tumor cells. J Cancer Metastasis Treat. (2024) 10:28. doi: 10.20517/2394-4722.2024.78

[28] Liu H Dilger JP . Different strategies for cancer treatment: targeting cancer cells or their neighbors? Chin J Cancer Res. (2025) 37:289. doi: 10.21147/j.issn.1000-9604.2025.02.12, PMID: 40353083 PMC12062981

[29] Baas P Scherpereel A Nowak AK Fujimoto N Peters S Tsao AS . First-line nivolumab plus ipilimumab in unresecta ble Malignant pleural mesothelioma (CheckMate 743): a multicentre, randomised, open-label, phase 3 trial. Lancet. (2021) 397:375–86. doi: 10.1016/S0140-6736(20)32714-8, PMID: 33485464

[30] Narote S Desai SA Patel VP Deshmukh R Raut N Dapse S . Identification of new immune target and signaling for cancer immunotherapy. Cancer Genet. (2025). 294-295:57–75. doi: 10.1016/j.cancergen.2025.03.004, PMID: 40154216

[31] Joshi RM Telang B Soni G Khalife A . Overview of perspectives on cancer, newer therapies, and future directions. Oncol Trans Med. (2024) 10:105–9. doi: 10.1097/ot9.0000000000000039

[32] Ren W Zhang H Li Y Sun W Peng H Guo H . Efficacy and safety of PD-1/PD-L1 inhibitors as first-line treatment for esophageal squamous cell carcinoma: a systematic review and meta-analysis. Front Immunol. (2025) 16:1563300. doi: 10.3389/fimmu.2025.1563300, PMID: 40207226 PMC11979238

