# Supplementary material for: Comparative safety profiles of first-line immunotherapy regimens in advanced esophageal squamous cell carcinoma: a network meta-analysis focusing on toxicity stratification
Source: Front Oncol. 2026 Jan 2;15:1700236. doi: 10.3389/fonc.2025.1700236 (PMC12807980; doi:10.3389/fonc.2025.1700236)
Supplement: Supplementary file 1 [file DataSheet1.docx]

Comparative Safety Profiles of First-Line Immunotherapy Regimens in Advanced Esophageal Squamous Cell Carcinoma: A Network Meta-Analysis Focusing on Toxicity Stratification

| **Table of Contents** | | |
| --- | --- | --- |
| Title | Content | page |
| Table S1 | PRISMA NMA Checklist of Items to Include When Reporting a Systematic Review Involving a Network Meta-analysis | 3-6 |
| Table S2 | Literature Search Strategy | 6-7 |
| Table S3 | Table S3. SUCRA-Based Ranking of Treatment Regimens for Safety Endpoints in the Bayesian Network Meta-Analysis | 8-9 |
| Table S4 | Model Fit Statistics for Random and Fixed Effects in Bayesian Network Meta-Analysis Across Safety Endpoints | 10 |
| Table S5 | Consistency and Inconsistency Model Fit Comparison and Heterogeneity Assessment Across Safety Endpoints in the Bayesian Network Meta-Analysis | 10 |
| Figure S1 | Forest Plot Comparing Grade ≥3 trAEsBetween ICIs Combination Therapy and Chemotherapy in Advanced ESCC | 11 |
| Figure S2 | Forest Plot Comparing Any irAEs Between ICIs Combination Therapy and Chemotherapy in Advanced ESCC | 11 |
| Figure S3 | Forest Plot Comparing Grade ≥3 irAEs Between ICIs Combination Therapy and Chemotherapy in Advanced ESCC | 11 |
| Figure S4 | Forest Plot Comparing Immune-mediated rash Between ICIs Combination Therapy and Chemotherapy in Advanced ESCC | 12 |
| Figure S5 | Forest Plot Comparing Immune-mediated hypothyroidism Between ICIs Combination Therapy and Chemotherapy in Advanced ESCC | 12 |
| Figure S6 | Forest Plot Comparing Immune-mediated hyperthyroidism Between ICIs Combination Therapy and Chemotherapy in Advanced ESCC | 12 |
| Figure S7 | Forest Plot Comparing Immune-mediated Pneumonitis Between ICIs Combination Therapy and Chemotherapy in Advanced ESCC | 12 |
| Figure S8 | Rank Probability Heatmap of Treatment Regimens for Grade ≥3 trAEs in Advanced ESCC | 13 |
| Figure S9 | Rank Probability Heatmap of Treatment Regimens for Any irAEs in Advanced ESCC | 14 |
| Figure S10 | Rank Probability Heatmap of Treatment Regimens for Grade ≥3 irAEs in Advanced ESCC | 14 |
| Figure S11 | Rank Probability Heatmap of Treatment Regimens for Immune-mediated rash in Advanced ESCC | 15 |
| Figure S12 | Rank Probability Heatmap of Treatment Regimens for Immune-mediated hypothyroidism in Advanced ESCC | 15 |
| Figure S13 | Rank Probability Heatmap of Treatment Regimens for Immune-mediated hyperthyroidism in Advanced ESCC | 16 |
| Figure S14 | Rank Probability Heatmap of Treatment Regimens for Immune-mediated Pneumonitis in Advanced ESCC | 16 |
| Figure S15 | Convergence Diagnostics of Bayesian Network Meta-Analysis for Grade ≥3 trAEs in Advanced ESCC | 17 |
| Figure S16 | Convergence Diagnostics of Bayesian Network Meta-Analysis for Any irAEs in Advanced ESCC | 18 |
| Figure S17 | Convergence Diagnostics of Bayesian Network Meta-Analysis for Grade ≥3 irAEs in Advanced ESCC | 19 |
| Figure S18 | Convergence Diagnostics of Bayesian Network Meta-Analysis for Immune-mediated rash in Advanced ESCC | 20 |
| Figure S19 | Convergence Diagnostics of Bayesian Network Meta-Analysis for Immune-mediated hypothyroidism in Advanced ESCC | 21 |
| Figure S20 | Convergence Diagnostics of Bayesian Network Meta-Analysis for Immune-mediated hyperthyroidism in Advanced ESCC | 22 |
| Figure S21 | Convergence Diagnostics of Bayesian Network Meta-Analysis for Immune-mediated Pneumonitis in Advanced ESCC | 23 |
| Figure S22 | Trace and Posterior Density Plots for Bayesian Network Meta-Analysis of Grade ≥3 Adverse Events in Advanced ESCC | 24 |
| Figure S23 | Trace and Posterior Density Plots for Bayesian Network Meta-Analysis of Any irAEs in Advanced ESCC | 25 |
| Figure S24 | Trace and Posterior Density Plots for Bayesian Network Meta-Analysis of Grade ≥3 irAEs in Advanced ESCC | 26 |
| Figure S25 | Trace and Posterior Density Plots for Bayesian Network Meta-Analysis of Immune-mediated rash in Advanced ESCC | 27 |
| Figure S26 | Trace and Posterior Density Plots for Bayesian Network Meta-Analysis of Immune-mediated hypothyroidism in Advanced ESCC | 28 |
| Figure S27 | Trace and Posterior Density Plots for Bayesian Network Meta-Analysis of Immune-mediated hyperthyroidism in Advanced ESCC | 29 |
| Figure S28 | Trace and Posterior Density Plots for Bayesian Network Meta-Analysis of Immune-mediated Pneumonitis in Advanced ESCC | 30 |
| Figure S29 | Funnel Plot for Assessment of Publication Bias in Grade ≥3 Adverse Events Among Advanced ESCC Patients Receiving ICI-Based Combination Therapy Versus Chemotherapy | 31 |
| Figure S30 | Funnel Plot for Assessment of Publication Bias in Any irAEs Among Advanced ESCC Patients Receiving ICI-Based Combination Therapy Versus Chemotherapy | 31 |
| Figure S31 | Funnel Plot for Assessment of Publication Bias in Grade ≥3 irAEs Among Advanced ESCC Patients Receiving ICI-Based Combination Therapy Versus Chemotherapy | 32 |
| Figure S32 | Funnel Plot for Assessment of Publication Bias in Immune-mediated rash Among Advanced ESCC Patients Receiving ICI-Based Combination Therapy Versus Chemotherapy | 32 |
| Figure S33 | Funnel Plot for Assessment of Publication Bias in Immune-mediated hypothyroidism  Among Advanced ESCC Patients Receiving ICI-Based Combination Therapy Versus Chemotherapy | 33 |
| Figure S34 | Funnel Plot for Assessment of Publication Bias in Immune-mediated hyperthyroidism  Among Advanced ESCC Patients Receiving ICI-Based Combination Therapy Versus Chemotherapy | 33 |
| Figure S35 | Funnel Plot for Assessment of Publication Bias in Immune-mediated Pneumonitis Among Advanced ESCC Patients Receiving ICI-Based Combination Therapy Versus Chemotherapy | 34 |
| Table S7 | network meta-regression adjusted for chemotherapy regimen | 34 |
| Table S8 | Definitions and Harmonization of Safety Endpoints Across Included RCTs | 35 |
| Table S9 | Raw Event Counts for Grade ≥3 trAEs, Any-Grade irAEs, and Grade ≥3 irAEs Across Included Trials | 36 |
| Table S10 | Raw Event Counts for Organ-Specific Immune-Related Adverse Events Across Included Trials | 36 |

Table S1 PRISMA NMA Checklist of Items to Include When Reporting a Systematic Review Involving a Network Meta-analysis

| **Section/Topic** | **Item #** | **Checklist Item** | **Reported on Page #** |
| --- | --- | --- | --- |
| **TITLE** |  |  |  |
| Title | 1 | Identify the report as a systematic review *incorporating*  *anetwork meta-analysis (or related form of meta-analysis).* | **1** |
|  |  |  |  |
| **ABSTRACT** |  |  |  |
| Structured summary | 2 | Provide a structured summary including, as applicable:  **Background:** main objectives  **Methods:** data sources; study eligibility criteria, participants, and interventions; study appraisal; and *synthesis methods, such as network meta-analysis.*  **Results:** number of studies and participants identified; summary estimates with corresponding confidence/credible intervals; *treatment rankings may also be discussed. Authors may choose to summarize pairwise comparisons against a chosen treatment included in their analyses for brevity.*  **Discussion/Conclusions:** limitations; conclusions and implications of findings.  **Other:** systematic review registration number with registry name. | 1-2 |
|  |  |  |  |
| **INTRODUCTION** |  |  |  |
| Rationale | 3 | Describe the rationale for the review in the context of what is already known*, including mention of why a network meta-analysis has been conducted.* | **2** |
| Objectives | 4 | Provide an explicit statement of questions being addressed, with reference to participants, interventions, comparisons, outcomes, and study design (PICOS). | 2-3 |
|  |  |  |  |
| **METHODS** |  |  |  |
| Protocol and registration | 5 | Indicate whether a review protocol exists and if and where it can be accessed (e.g., Web address); and, if available, provide registration information, including registration number. | 3 |
| Eligibility criteria | 6 | Specify study characteristics (e.g., PICOS, length of follow-up) and report characteristics (e.g., years considered, language, publication status) used as criteria for eligibility, giving rationale. *Clearly describe eligible treatments included in the treatment network, and note whether any have been clustered or merged into the same node (with justification).* | 3-4 |
| Information sources | 7 | Describe all information sources (e.g., databases with dates of coverage, contact with study authors to identify additional studies) in the search and date last searched. | 3 |
| Search | 8 | Present full electronic search strategy for at least one database, including any limits used, such that it could be repeated. | 3，Supplementary  TableS2 |
| Study selection | 9 | State the process for selecting studies (i.e., screening, eligibility, included in systematic review, and, if applicable, included in the meta-analysis). | **3** |
| Data collection process | 10 | Describe method of data extraction from reports (e.g., piloted forms, independently, in duplicate) and any processes for obtaining and confirming data from investigators. | 4 |
| Data items | 11 | List and define all variables for which data were sought (e.g., PICOS, funding sources) and any assumptions and simplifications made. | 4 |
| **Geometry of the network** | **S1** | Describe methods used to explore the geometry of the treatment network under study and potential biases related to it. This should include how the evidence base has been graphically summarized for presentation, and what characteristics were compiled and used to describe the evidence base to readers. | **4** |
| Risk of bias within individual studies | 12 | Describe methods used for assessing risk of bias of individual studies (including specification of whether this was done at the study or outcome level), and how this information is to be used in any data synthesis. | 4 |
| Summary measures | 13 | State the principal summary measures (e.g., risk ratio, difference in means). *Also describe the use of additional summary measures assessed, such as treatment rankings and surface under the cumulative ranking curve (SUCRA) values, as well as modified approaches used to present summary findings from meta-analyses.* | 4 |
| Planned methods of analysis | 14 | Describe the methods of handling data and combining results of studies for each network meta-analysis. This should include, but not be limited to:   - *Handling of multi-arm trials;* - *Selection of variance structure;* - *Selection of prior distributions in Bayesian analyses; and* - *Assessment of model fit.* | 4-5 |
| **Assessment of Inconsistency** | **S2** | Describe the statistical methods used to evaluate the agreement of direct and indirect evidence in the treatment network(s) studied. Describe efforts taken to address its presence when found. | 4 |
| Risk of bias across studies | 15 | Specify any assessment of risk of bias that may affect the cumulative evidence (e.g., publication bias, selective reporting within studies). | 4 |
| Additional analyses | 16 | Describe methods of additional analyses if done, indicating which were pre-specified. This may include, but not be limited to, the following:   - Sensitivity or subgroup analyses; - Meta-regression analyses; - *Alternative formulations of the treatment network; and* - *Use of alternative prior distributions for Bayesian analyses (if applicable).* | 4-5 |
| **RESULTS†** |  |  |  |
| Study selection | 17 | Give numbers of studies screened, assessed for eligibility, and included in the review, with reasons for exclusions at each stage, ideally with a flow diagram. | 5-6，Fig1 |
| **Presentation of network structure** | **S3** | Provide a network graph of the included studies to enable visualization of the geometry of the treatment network. | 13,Fig4 |
| **Summary of network geometry** | **S4** | Provide a brief overview of characteristics of the treatment network. This may include commentary on the abundance of trials and randomized patients for the different interventions and pairwise comparisons in the network, gaps of evidence in the treatment network, and potential biases reflected by the network structure. | 12 |
| Study characteristics | 18 | For each study, present characteristics for which data were extracted (e.g., study size, PICOS, follow-up period) and provide the citations. | 5-6,Table 1 |
| Risk of bias within studies | 19 | Present data on risk of bias of each study and, if available, any outcome level assessment. | 10,Figure 2 |
| Results of individual studies | 20 | For all outcomes considered (benefits or harms), present, for each study: 1) simple summary data for each intervention group, and 2) effect estimates and confidence intervals. *Modified approaches may be needed to deal with information from larger networks.* | 11-16 |
| Synthesis of results | 21 | Present results of each meta-analysis done, including confidence/credible intervals. *In larger networks, authors may focus on comparisons versus a particular comparator (e.g. placebo or standard care), with full findings presented in an appendix. League tables and forest plots may be considered to summarize pairwise comparisons.* If additional summary measures were explored (such as treatment rankings), these should also be presented. | 11-19 |
| **Exploration for inconsistency** | **S5** | Describe results from investigations of inconsistency. This may include such information as measures of model fit to compare consistency and inconsistency models, *P* values from statistical tests, or summary of inconsistency estimates from different parts of the treatment network. | 18-19 |
| Risk of bias across studies | 22 | Present results of any assessment of risk of bias across studies for the evidence base being studied. | 18-19 |
| Results of additional analyses | 23 | Give results of additional analyses, if done (e.g., sensitivity or subgroup analyses, meta-regression analyses*, alternative network geometries studied, alternative choice of prior distributions for Bayesian analyses,* and so forth). | 18-19 |
|  |  |  |  |
| **DISCUSSION** |  |  |  |
| Summary of evidence | 24 | Summarize the main findings, including the strength of evidence for each main outcome; consider their relevance to key groups (e.g., healthcare providers, users, and policy-makers). | 19-20 |
| Limitations | 25 | Discuss limitations at study and outcome level (e.g., risk of bias), and at review level (e.g., incomplete retrieval of identified research, reporting bias). *Comment on the validity of the assumptions, such as transitivity and consistency. Comment on any concerns regarding network geometry (e.g., avoidance of certain comparisons).* | 19-20 |
| Conclusions | 26 | Provide a general interpretation of the results in the context of other evidence, and implications for future research. | 1 |
|  |  |  |  |
| **FUNDING** |  |  |  |
| Funding | 27 | Describe sources of funding for the systematic review and other support (e.g., supply of data); role of funders for the systematic review. This should also include information regarding whether funding has been received from manufacturers of treatments in the network and/or whether some of the authors are content experts with professional conflicts of interest that could affect use of treatments in the network. | NA |

PICOS = population, intervention, comparators, outcomes, study design.

* Text in italics indicate S wording specific to reporting of network meta-analyses that has been added to guidance from the PRISMA statement.

† Authors may wish to plan for use of appendices to present all relevant information in full detail for items in this section.

| **Table S2.Literature Search Strategy** | |
| --- | --- |
| **Pubmed** | ("Immune Checkpoint Inhibitors"[Mesh]  OR "Checkpoint Inhibitors, Immune"[Title/Abstract]  OR "Immune Checkpoint Inhibitor"[Title/Abstract]  OR "Immune Checkpoint Blockade"[Title/Abstract]  OR "PD-1 Inhibitor"[Title/Abstract]  OR "PD-L1 Inhibitor"[Title/Abstract]  OR "CTLA-4 Inhibitor"[Title/Abstract]  OR "Programmed Death-Ligand 1 Inhibitor"[Title/Abstract]  OR "Programmed Cell Death Protein 1 Inhibitor"[Title/Abstract])  AND  ("Esophageal Squamous Cell Carcinoma"[Mesh]  OR "Esophageal Squamous Cell Carcinoma"[Title/Abstract]  OR "Oesophageal Squamous Cell Carcinoma"[Title/Abstract])  AND  (randomized controlled trial[pt]  OR controlled clinical trial[pt]  OR randomized[Title/Abstract]  OR randomly[Title/Abstract]  OR trial[Title/Abstract]  OR placebo[Title/Abstract]  OR drug therapy[sh]  OR groups[Title/Abstract]) |
| **Web of Science** | TS=(("Esophageal Squamous Cell Carcinoma" OR  "Esophageal Squamous-Cell Carcinoma" OR  "Esophageal Squamous Cell Cancer" OR  "Oesophageal Squamous Cell Carcinoma" OR  "Oesophageal Squamous Cell Cancer" OR  "Oesophageal Cancer"))  AND  TS=(("Immune Checkpoint Inhibitor*" OR  "Checkpoint Inhibitor*" OR  "Immune Checkpoint Blocker*" OR  "Checkpoint Blockade" OR  "Immune Checkpoint Blockade" OR  "PD-1 Inhibitor*" OR  "PD-L1 Inhibitor*" OR  "CTLA-4 Inhibitor*" OR  "Programmed Death-Ligand 1 Inhibitor*" OR  "Programmed Cell Death Protein 1 Inhibitor*" OR  "PD-1/PD-L1 Blockade"))  AND  TS=("randomized controlled trial" OR  "controlled clinical trial" OR  randomized OR  randomised OR  randomly OR  placebo OR  sham OR  trial OR  groups) |
| **Cochrane** | #1 MeSH descriptor: [Esophageal Squamous Cell Carcinoma] explode all trees  #2 (esophageal OR oesophageal):ti,ab,kw  #3 ("squamous cell" OR squamous):ti,ab,kw  #4 (cancer OR carcinoma* OR neoplas*):ti,ab,kw  #5 #2 AND #3 AND #4  #6 #1 OR #5  #7 MeSH descriptor: [Immune Checkpoint Inhibitors] explode all trees  #8 ("immune checkpoint inhibitor*" OR  "checkpoint blockade" OR  "PD-1 inhibitor*" OR  "PD-L1 inhibitor*" OR  "CTLA-4 inhibitor*" OR  "programmed cell death protein 1 inhibitor*" OR  "programmed death-ligand 1 inhibitor*"  ):ti,ab,kw  #9 #7 OR #8  #10 #6 AND #9 |
| **Embase** | #1 'esophageal squamous cell carcinoma'/exp  #2 (esophageal OR oesophageal):ti,ab  #3 (squamous OR "squamous cell"):ti,ab  #4 (carcinoma* OR cancer OR neoplas*):ti,ab  #5 #2 AND #3 AND #4  #6 #1 OR #5  #7 'immune checkpoint inhibitor'/exp  #8 ("immune checkpoint inhibitor*" OR  "checkpoint blockade" OR  "PD-1 inhibitor*" OR  "PD-L1 inhibitor*" OR  "CTLA-4 inhibitor*" OR  "programmed death-ligand 1 inhibitor*" OR  "programmed cell death protein 1 inhibitor*"):ti,ab  #9 #7 OR #8  #10 ('randomized controlled trial'/de OR  'double-blind procedure'/de OR  'single-blind procedure'/de OR  'crossover procedure'/de OR  random*:ti,ab OR  trial:ti,ab OR  placebo*:ti,ab OR  (cross NEXT/1 over*):ti,ab OR  (doubl* NEAR/1 blind*):ti,ab OR  (singl* NEAR/1 blind*):ti,ab OR  assign*:ti,ab OR  allocat*:ti,ab OR  volunteer*:ti,ab)  #11 #6 AND #9 AND #10 |

**Table S3. SUCRA-Based Ranking of Treatment Regimens for Safety Endpoints in the Bayesian Network Meta-Analysis**

| **Endpoints** | **Drug** | **SUCRA**（%） | **Rank** | **Endpoints** | **Drug** | **SUCRA**（%） | **Rank** |
| --- | --- | --- | --- | --- | --- | --- | --- |
| Grade ≥3 trAEs | Camre-chemo | 87.8 | 1 | Any irAEs | Chemo | 99.9 | 1 |
|  | Nivo-ipi | 86.4 | 2 |  | Toripa-chemo | 83.8 | 2 |
|  | Chemo | 68.7 | 3 |  | Serplu-chemo | 65.5 | 3 |
|  | Tisle-chemo | 53.2 | 4 |  | Sinti-chemo | 58.4 | 4 |
|  | Sugema-chemo | 48.5 | 5 |  | Sugema-chemo | 49.4 | 5 |
|  | Serplu-chemo | 42.2 | 6 |  | Camre-chemo | 22.2 | 6 |
|  | Sinti-chemo | 35 | 7 |  | Nivo-chemo | 20.7 | 7 |
|  | Toripa-chemo | 20.3 | 8 |  | Nivo-ipi | 0 | 8 |
|  |  |  |  |  | Chemo | 99.9 | 9 |
| Grade ≥3 irAEs | Chemo | 97.7 | 1 | Immune-mediated hypothyroidism | Chemo | 97.7 | 1 |
|  | Camre-chemo | 71.6 | 2 |  | Toripa-chemo | 69.8 | 2 |
|  | Nivo-chemo | 69.3 | 3 |  | Sinti-chemo | 65 | 3 |
|  | Sugema-chemo | 43.5 | 4 |  | Nivo-chemo | 62.6 | 4 |
|  | Serplu-chemo | 38.2 | 5 |  | Camre-chemo | 43.6 | 5 |
|  | Toripa-chemo | 29.7 | 6 |  | Sugema-chemo | 28.8 | 6 |
|  | Nivo-ipi | 25 | 7 |  | Serplu-chemo | 18.5 | 7 |
|  | Sinti-chemo | 24.9 | 8 |  | Nivo-ipi | 14 | 8 |
| Immune-mediated hyperthyroidism | Chemo | 93.5 | 1 | Immune-mediated pneumonitis | Sinti-chemo | 79.6 | 1 |
|  | Serplu-chemo | 65.5 | 2 |  | Chemo | 77.6 | 2 |
|  | Sugema-chemo | 52.5 | 3 |  | Serplu-chemo | 63.6 | 3 |
|  | Sinti-chemo | 35.7 | 4 |  | Sugema-chemo | 61.9 | 4 |
|  | Toripa-chemo | 27.7 | 5 |  | Camre-chemo | 53.6 | 5 |
|  | Camre-chemo | 25.1 | 6 |  | Toripa-chemo | 43.7 | 6 |
|  |  |  |  |  | Nivo-chemo | 16.2 | 7 |
|  |  |  |  |  | Nivo-ipi | 3.8 | 7 |
| Immune-mediated rash | Chemo | 98.7 | 1 |  |  |  |  |
|  | Serplu-chemo | 72.7 | 2 |  |  |  |  |
|  | Camre-chemo | 64.2 | 3 |  |  |  |  |
|  | Toripa-chemo | 60 | 4 |  |  |  |  |
|  | Nivo-chemo | 40.5 | 5 |  |  |  |  |
|  | Sinti-chemo | 31.5 | 6 |  |  |  |  |
|  | Sugema-chemo | 24.8 | 7 |  |  |  |  |
|  | Nivo-ipi | 7.7 | 8 |  |  |  |  |

**Table S4. Model Fit Statistics for Random and Fixed Effects in Bayesian Network Meta-Analysis Across Safety Endpoints**

| **Endpoints** | **Effect Model** | **Dbar** | **pD** | **DIC** |
| --- | --- | --- | --- | --- |
| Grade ≥3 trAEs | Random effect | 14.96 | 14.95 | 29.91 |
|  | Fixed effect | 14.96 | 14.95 | 29.91 |
| Any irAEs | Random effect | 12.98 | 12.85 | 25.83 |
|  | Fixed effect | 12.99 | 12.86 | 25.85 |
| Grade ≥3 irAEs | Random effect | 13.25 | 12.08 | 25.33 |
|  | Fixed effect | 13.33 | 12.11 | 25.44 |
| Immune-mediated  hypothyroidism | Random effect | 13.15 | 12.49 | 25.64 |
|  | Fixed effect | 13.12 | 12.51 | 25.63 |
| Immune-mediated  hyperthyroidism | Random effect | 11.25 | 8.49 | 19.74 |
|  | Fixed effect | 11.24 | 8.5 | 19.74 |
| Immune-mediated  rash | Random effect | 13.37 | 12.18 | 25.55 |
|  | Fixed effect | 13.35 | 12.14 | 25.49 |
| Immune-mediated  pneumonitis | Random effect | 12.52 | 10.78 | 23.30 |
|  | Fixed effect | 12.46 | 10.71 | 23.17 |

**Table S5. Consistency and Inconsistency Model Fit Comparison and Heterogeneity Assessment Across Safety Endpoints in the Bayesian Network Meta-Analysis**

| **Endpoints** | **Model Type** | **Dbar** | **pD** | **DIC** | **I^2^** |
| --- | --- | --- | --- | --- | --- |
| Grade ≥3 trAEs | Consistency | 15.03 | 15.03 | 30.06 | 7% |
|  | Inconsistency | 14.93 | 14.92 | 29.85 | 6% |
| Any irAEs | Consistency | 13.03 | 13.03 | 26.06 | 8% |
|  | Inconsistency | 12.96 | 12.84 | 25.80 | 7% |
| Grade ≥3 irAEs | Consistency | 13.3 | 13.3 | 26.6 | 10% |
|  | Inconsistency | 13.31 | 12.13 | 25.44 | 10% |
| Immune-mediated  hypothyroidism | Consistency | 13.14 | 13.14 | 26.28 | 9% |
|  | Inconsistency | 13.14 | 12.53 | 25.67 | 9% |
| Immune-mediated  hyperthyroidism | Consistency | 9.38 | 9.3 | 18.68 | 4% |
|  | Inconsistency | 11.28 | 8.49 | 19.77 | 20% |
| Immune-mediated  rash | Consistency | 13.37 | 13.37 | 26.74 | 10% |
|  | Inconsistency | 13.25 | 12.05 | 25.3 | 9% |
| Immune-mediated  pneumonitis | Consistency | 13.54 | 13.54 | 27.08 | 12% |
|  | Inconsistency | 13.55 | 11.77 | 25.32 | 12% |

**
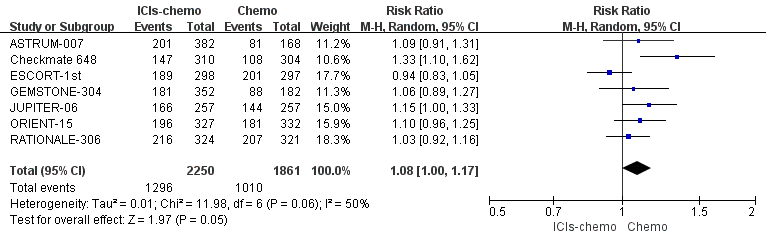
**

**Figure S1.Forest Plot Comparing Grade ≥3 trAEs Between ICIs Combination Therapy and Chemotherapy in Advanced ESCC**


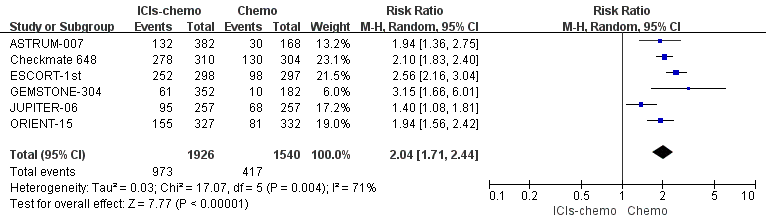


**Figure S2. Forest Plot Comparing Any irAEs Between ICIs Combination Therapy and Chemotherapy in Advanced ESCC**

**
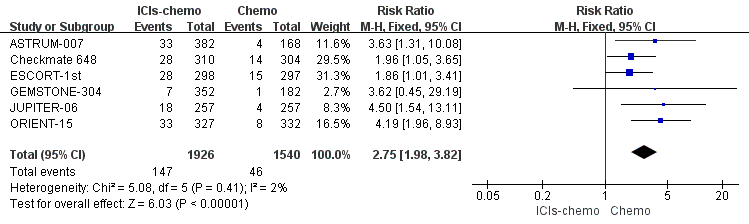
**

**Figure S3. Forest Plot Comparing Grade ≥3 irAEs Between ICIs Combination Therapy and Chemotherapy in Advanced ESCC**

**
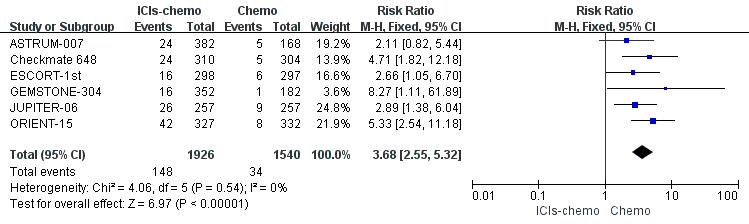
**

**Figure S4. Forest Plot Comparing Immune-mediated rash Between ICIs Combination Therapy and Chemotherapy in Advanced ESCC**

**
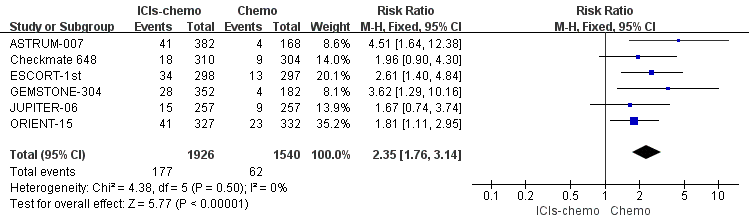
**

**Figure S5. Forest Plot Comparing Immune-mediated hypothyroidism Between ICIs Combination Therapy and Chemotherapy in Advanced ESCC**

**
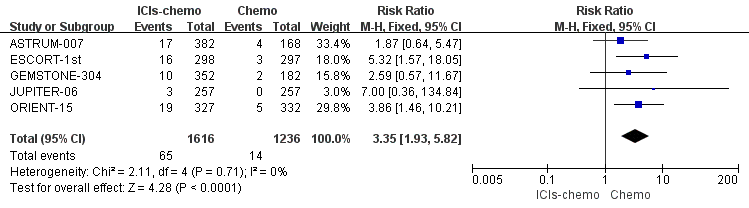
**

**Figure S6. Forest Plot Comparing Immune-mediated hyperthyroidism Between ICIs Combination Therapy and Chemotherapy in Advanced ESCC**

**
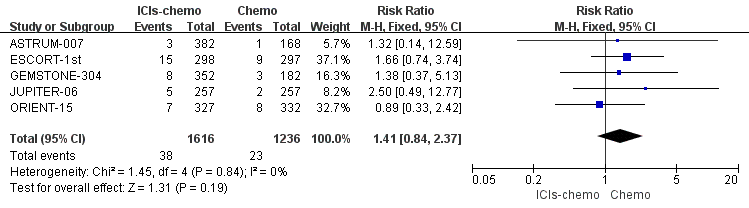
**

**Figure S7. Forest Plot Comparing Immune-mediated Pneumonitis Between ICIs Combination Therapy and Chemotherapy in Advanced ESCC**


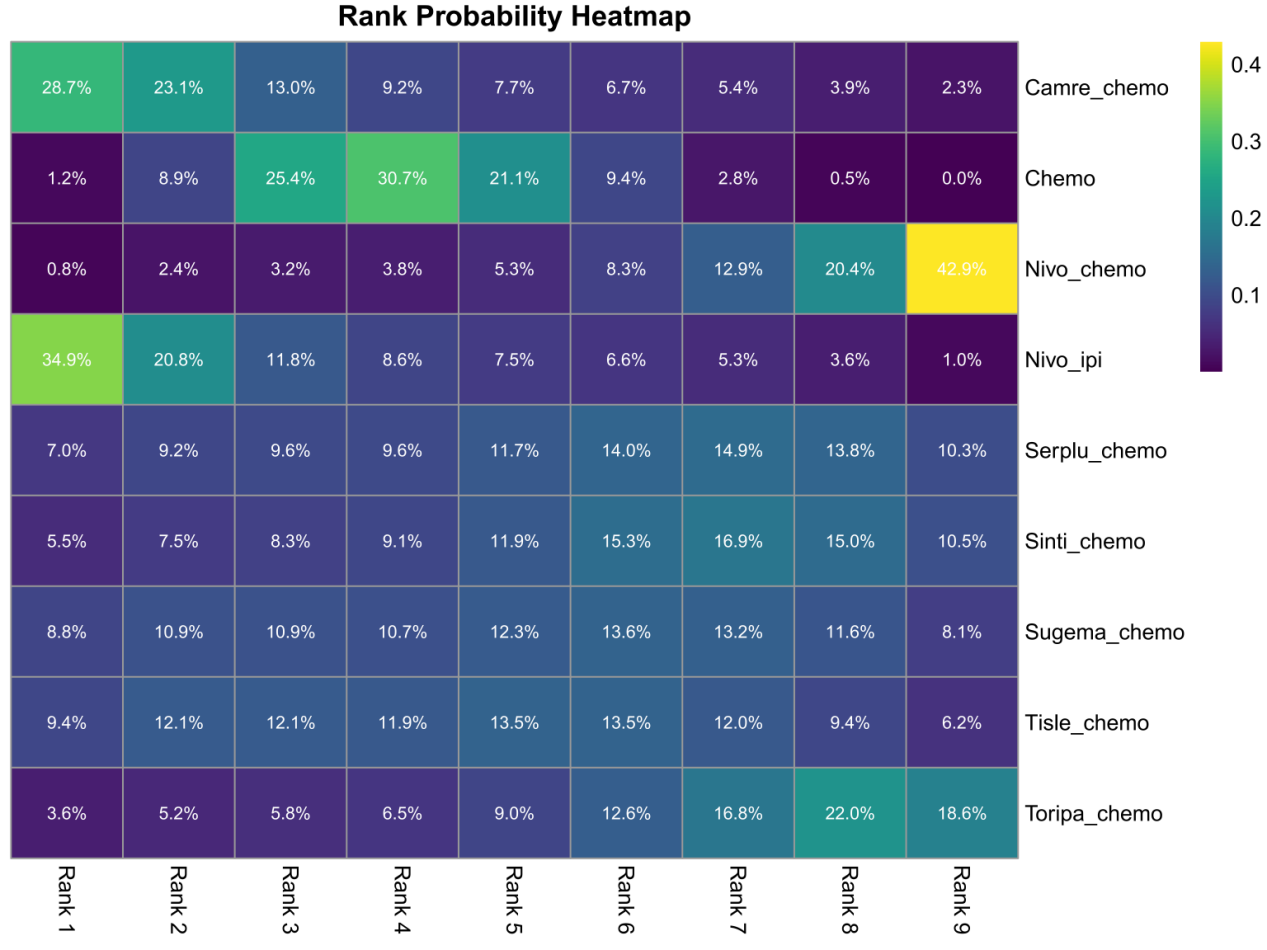


**Figure S8. Rank Probability Heatmap of Treatment Regimens for Grade ≥3 trAEs in Advanced ESCC
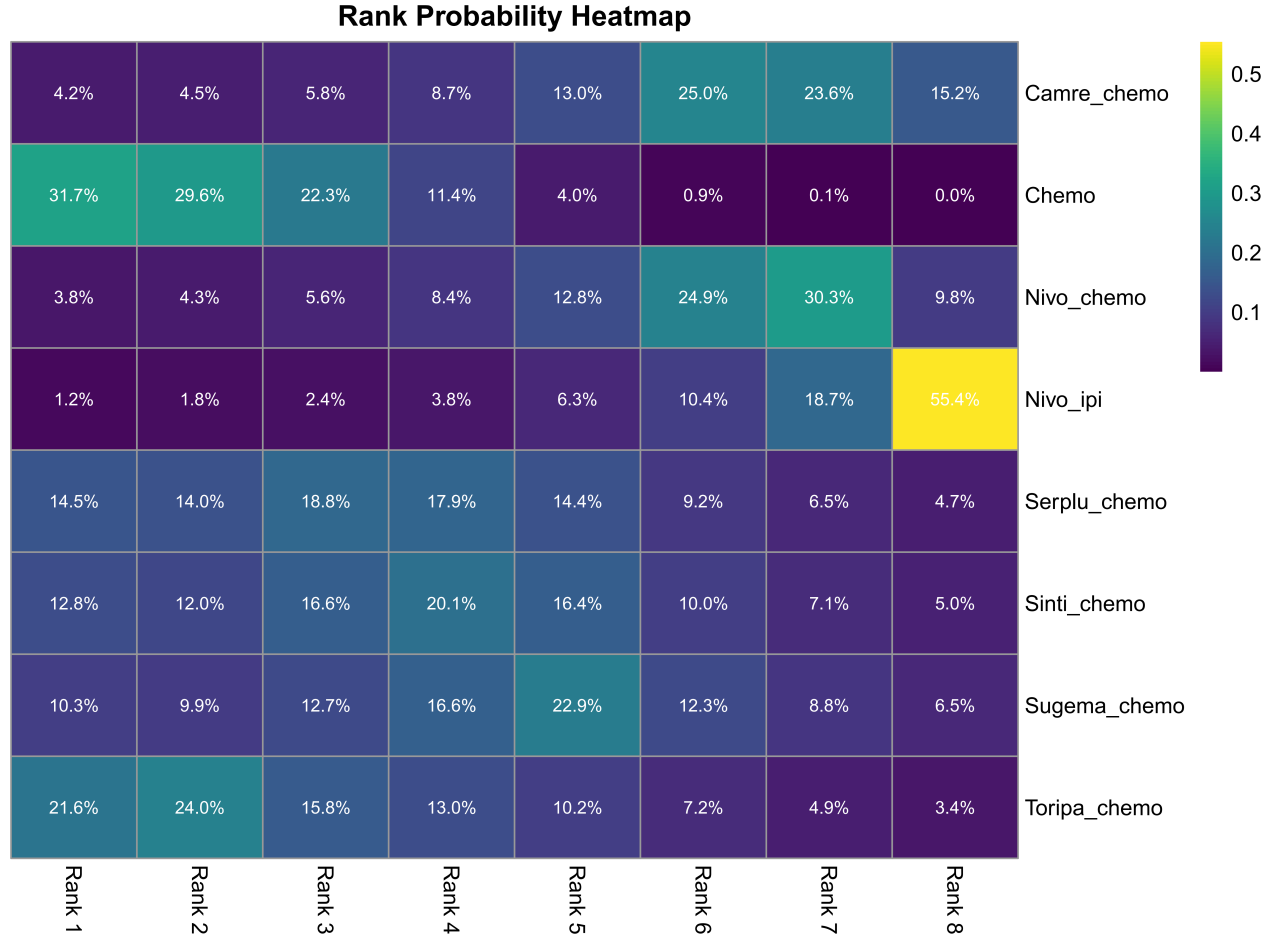
**

**Figure S9. Rank Probability Heatmap of Treatment Regimens for Any irAEs in Advanced ESCC**


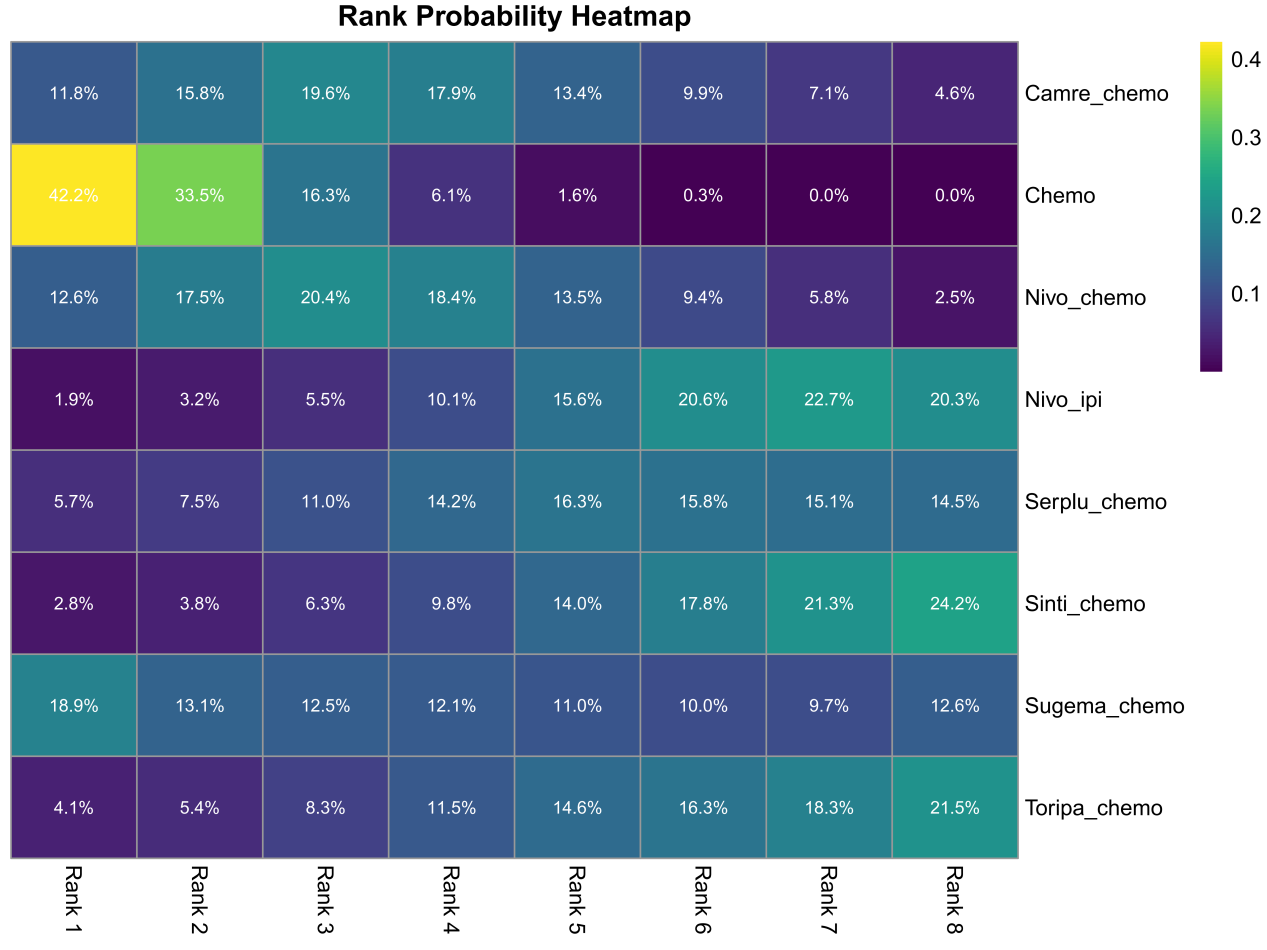


**Figure S10. Rank Probability Heatmap of Treatment Regimens for Grade ≥3 irAEs in Advanced ESCC**

**
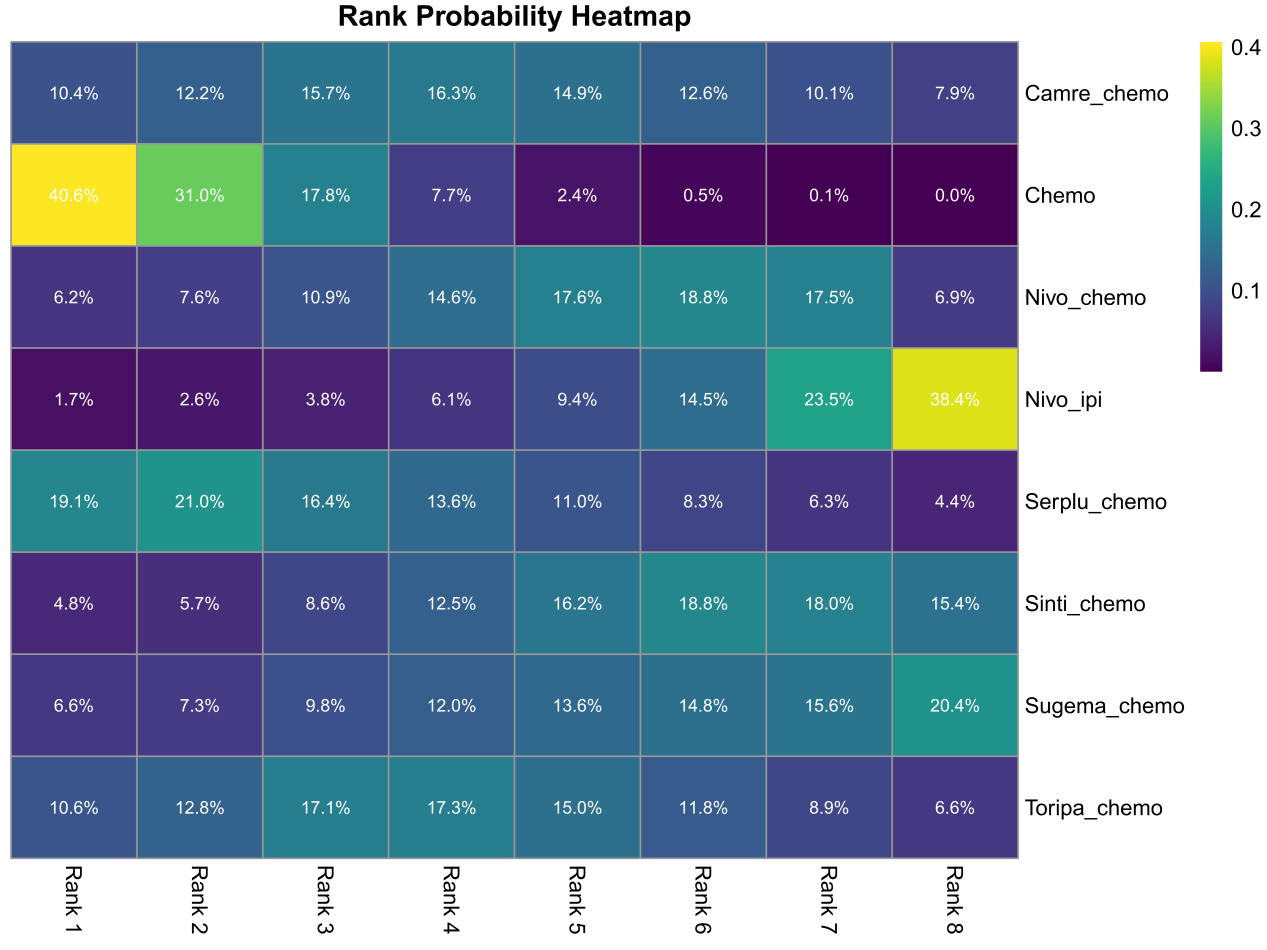
**

**Figure S11. Rank Probability Heatmap of Treatment Regimens for Immune-mediated rash in Advanced ESCC**


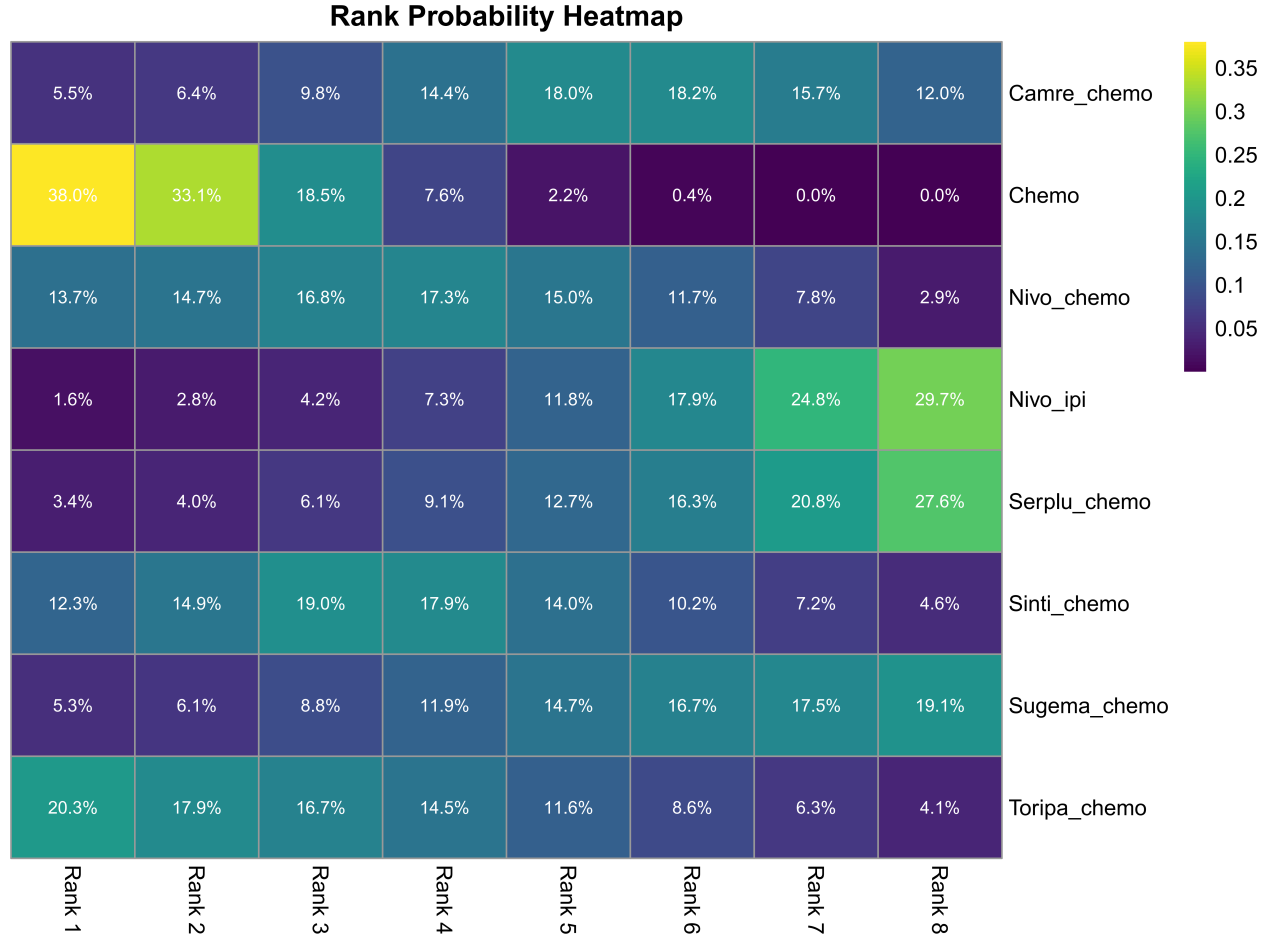


**Figure S12. Rank Probability Heatmap of Treatment Regimens for Immune-mediated hypothyroidism in Advanced ESCC**


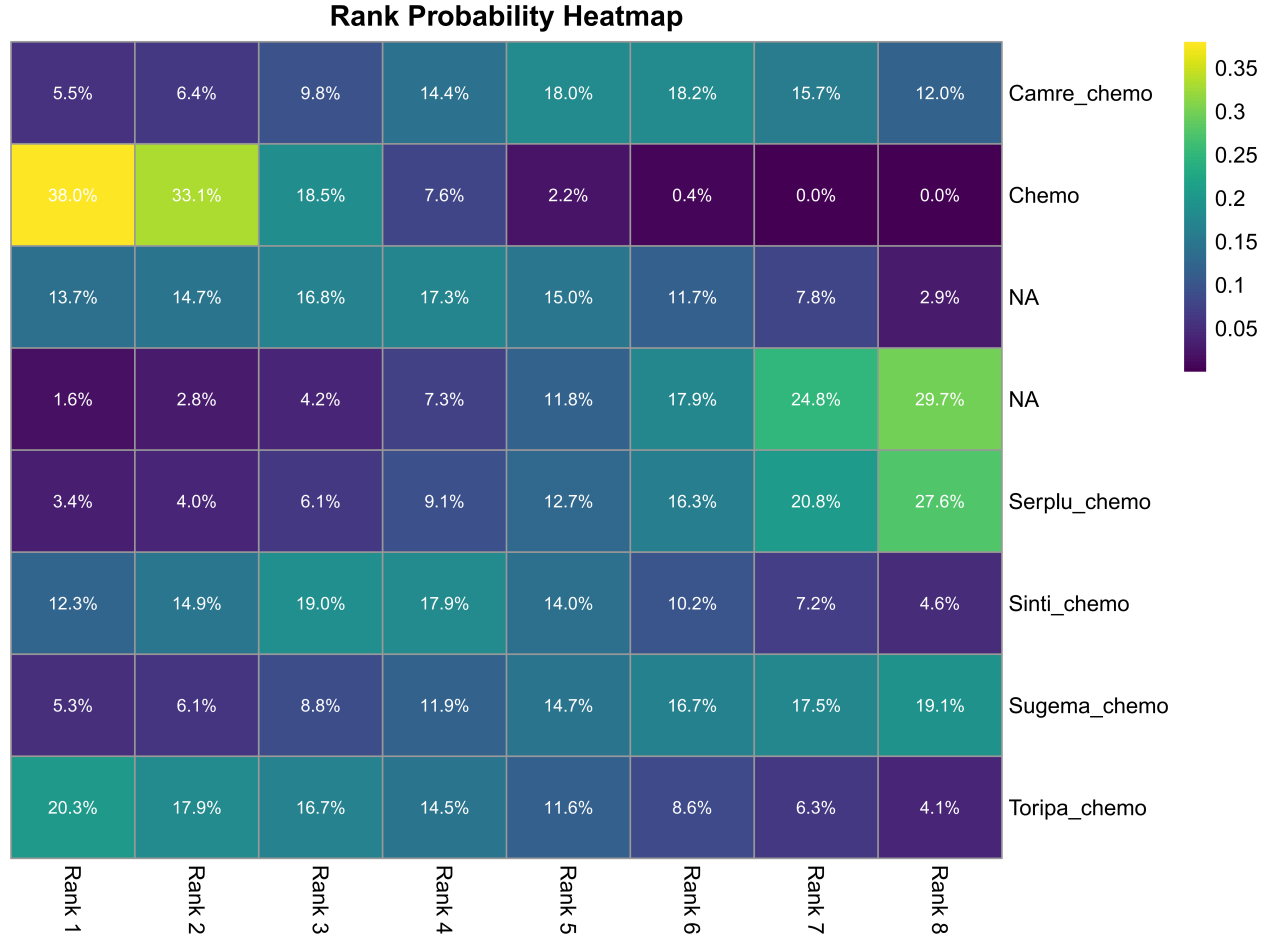


**Figure S13. Rank Probability Heatmap of Treatment Regimens for Immune-mediated hyperthyroidism in Advanced ESCC**

**
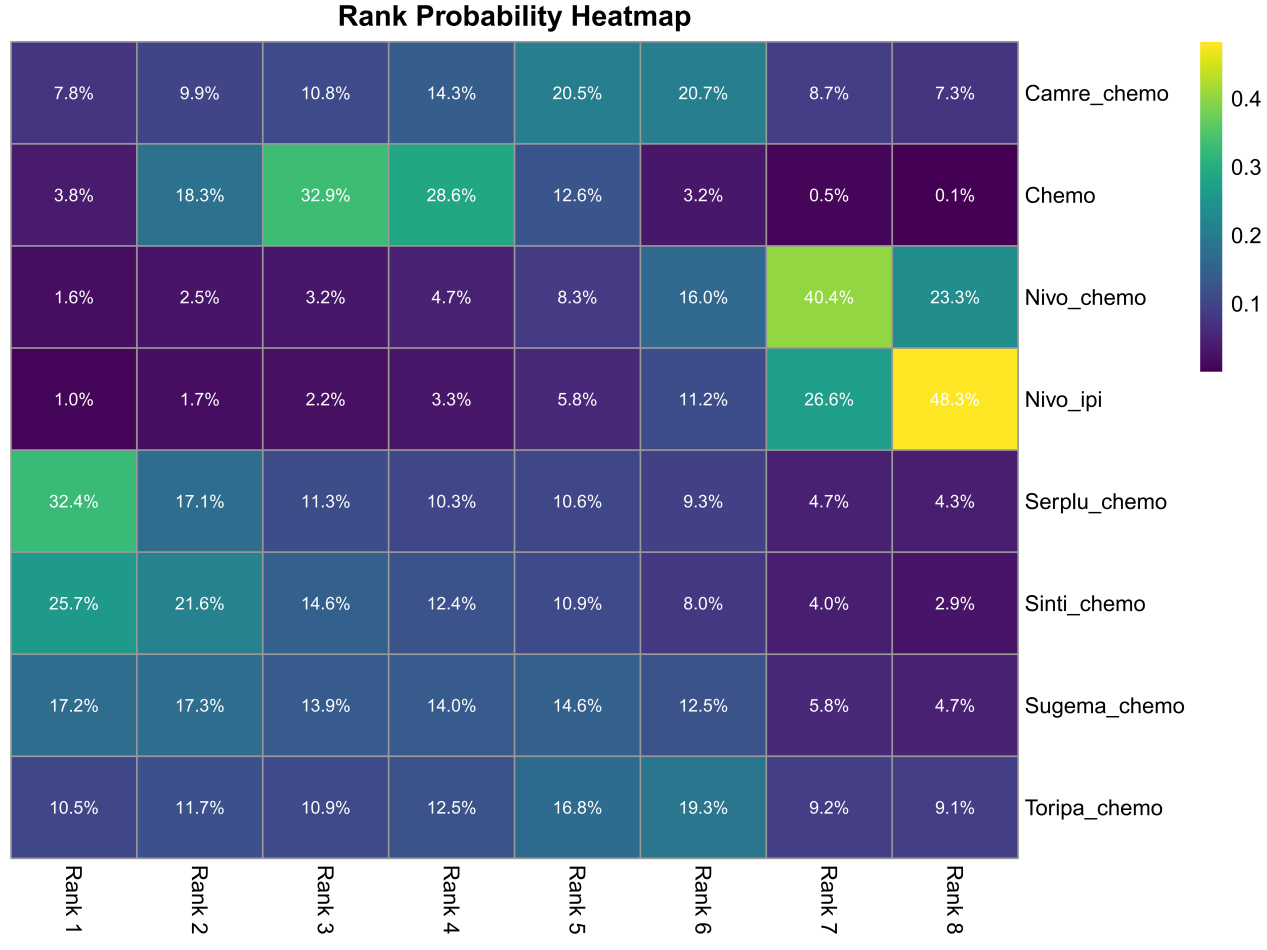
**

**Figure S14. Rank Probability Heatmap of Treatment Regimens for Immune-mediated Pneumonitis in Advanced ESCC**

**
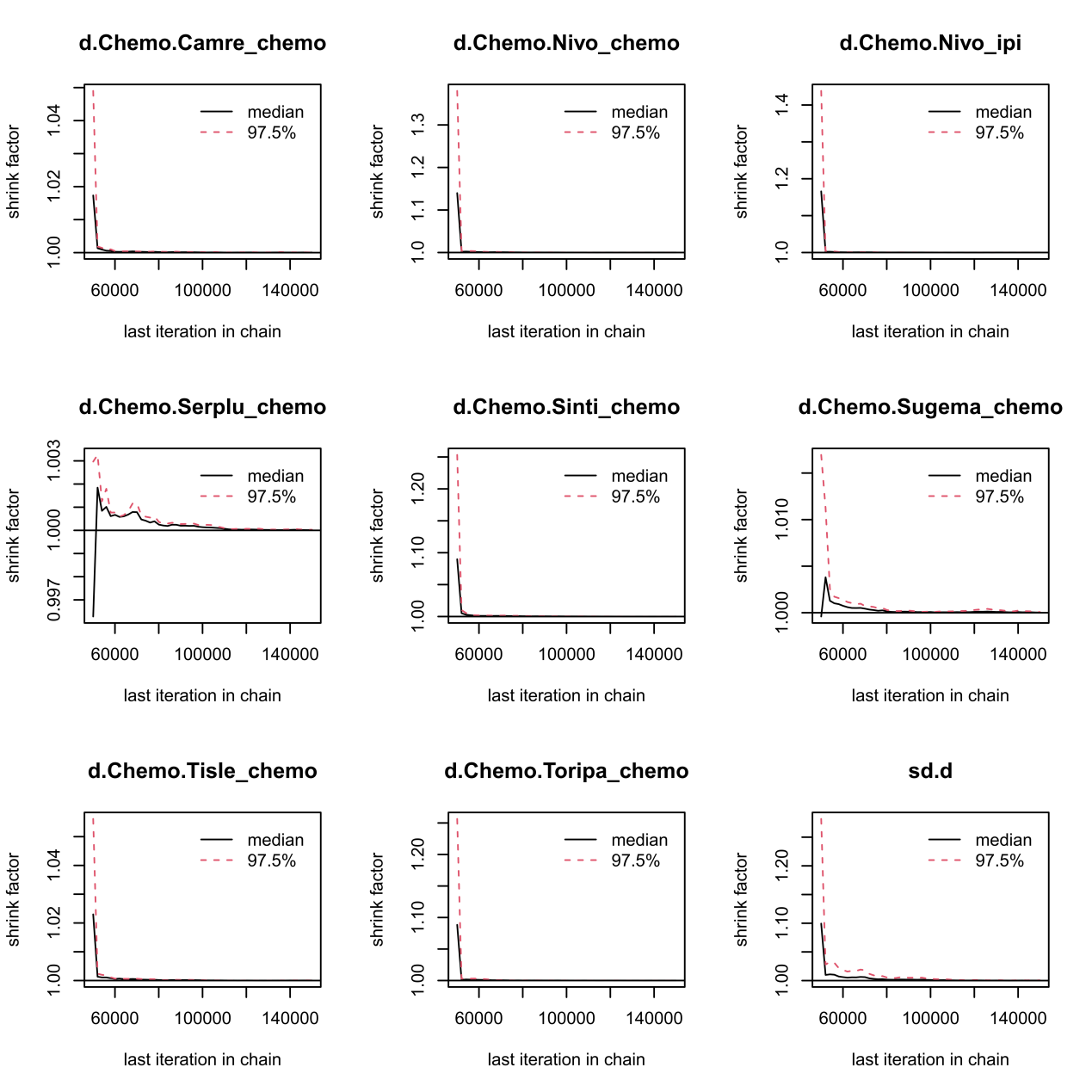
**

**Figure S15.Convergence Diagnostics of Bayesian Network Meta-Analysis for Grade ≥3 trAEs in Advanced ESCC**

**
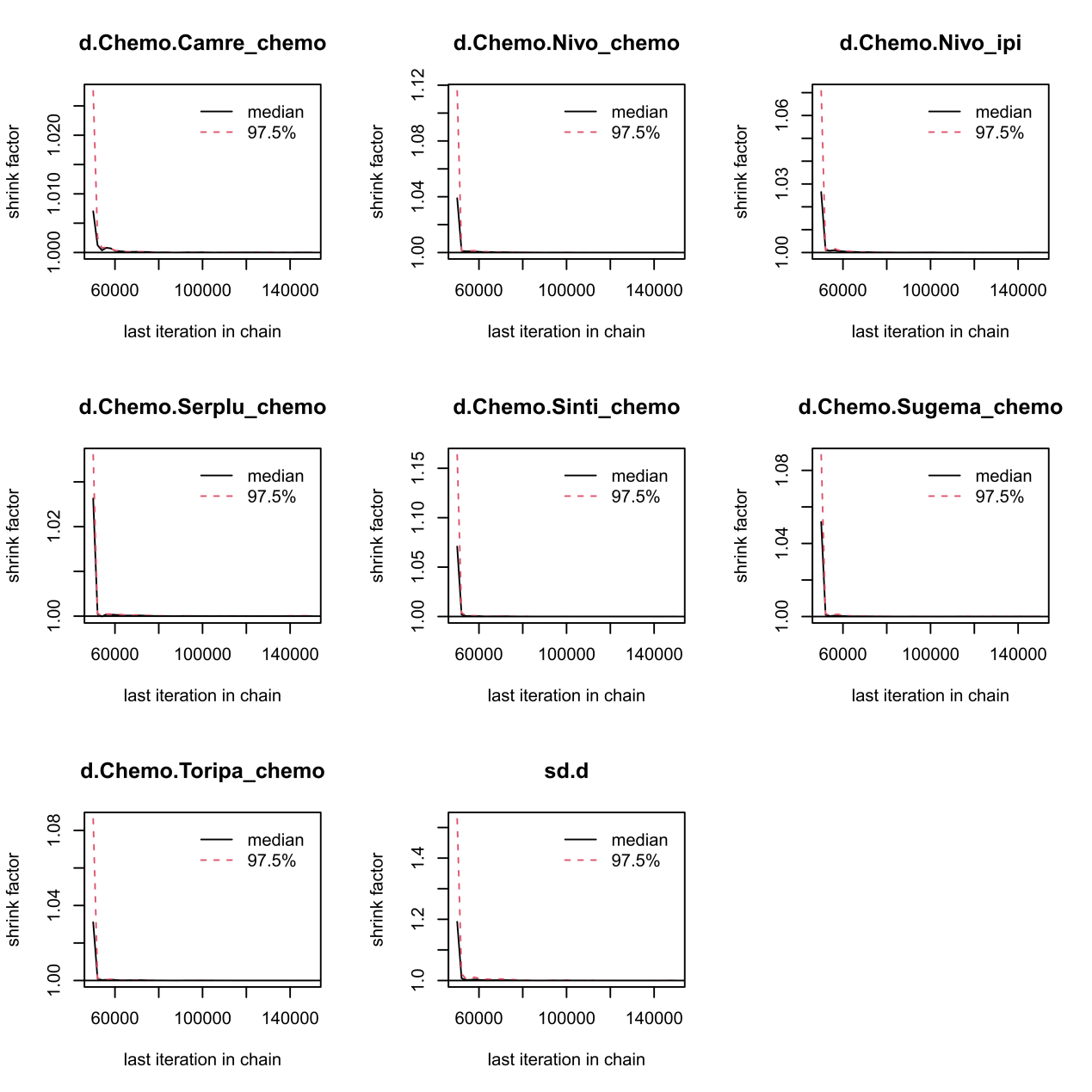
**

**Figure S16.Convergence Diagnostics of Bayesian Network Meta-Analysis for Any irAEs in Advanced ESCC**

**
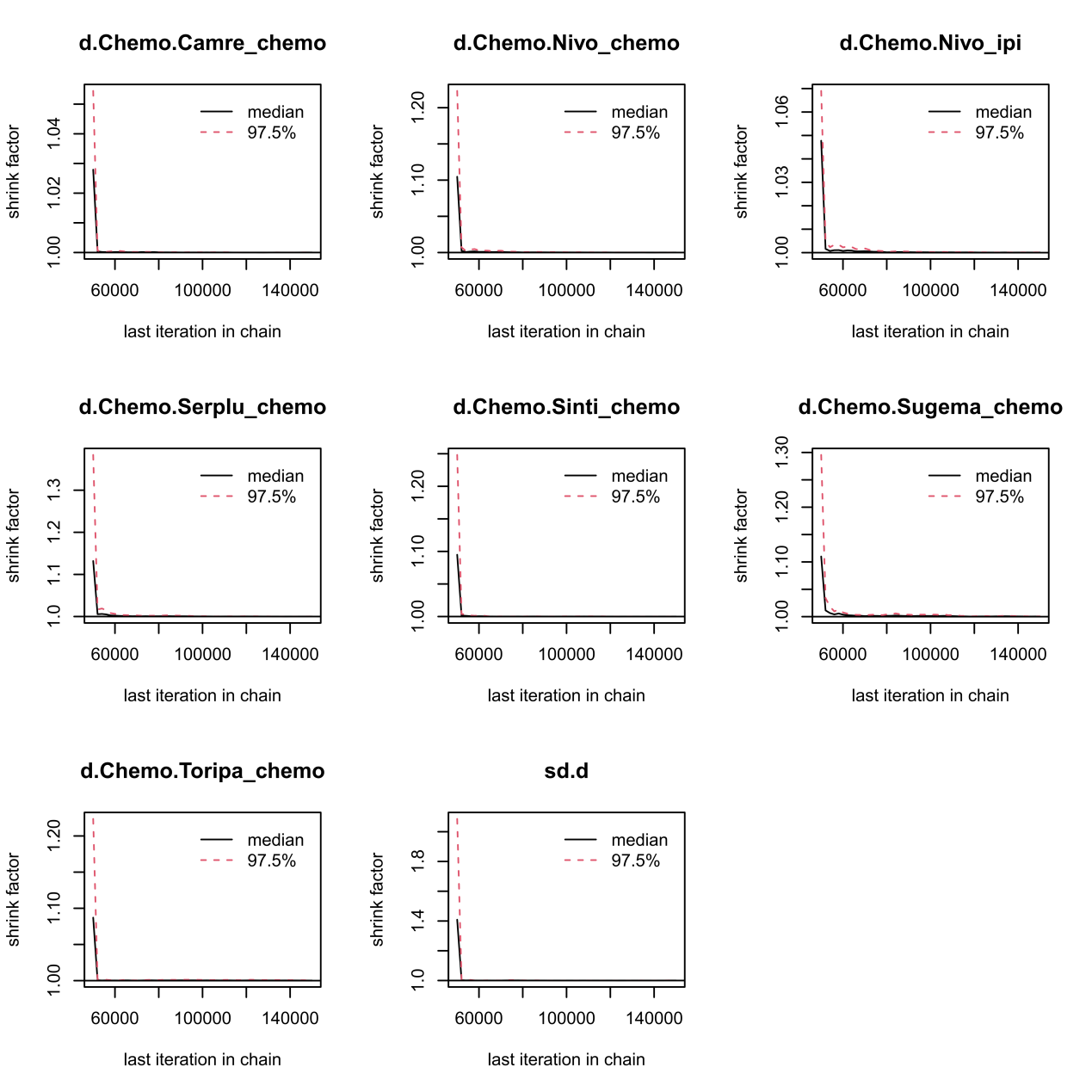
**

**Figure S17.Convergence Diagnostics of Bayesian Network Meta-Analysis for Grade ≥3 irAEs in Advanced ESCC**

**
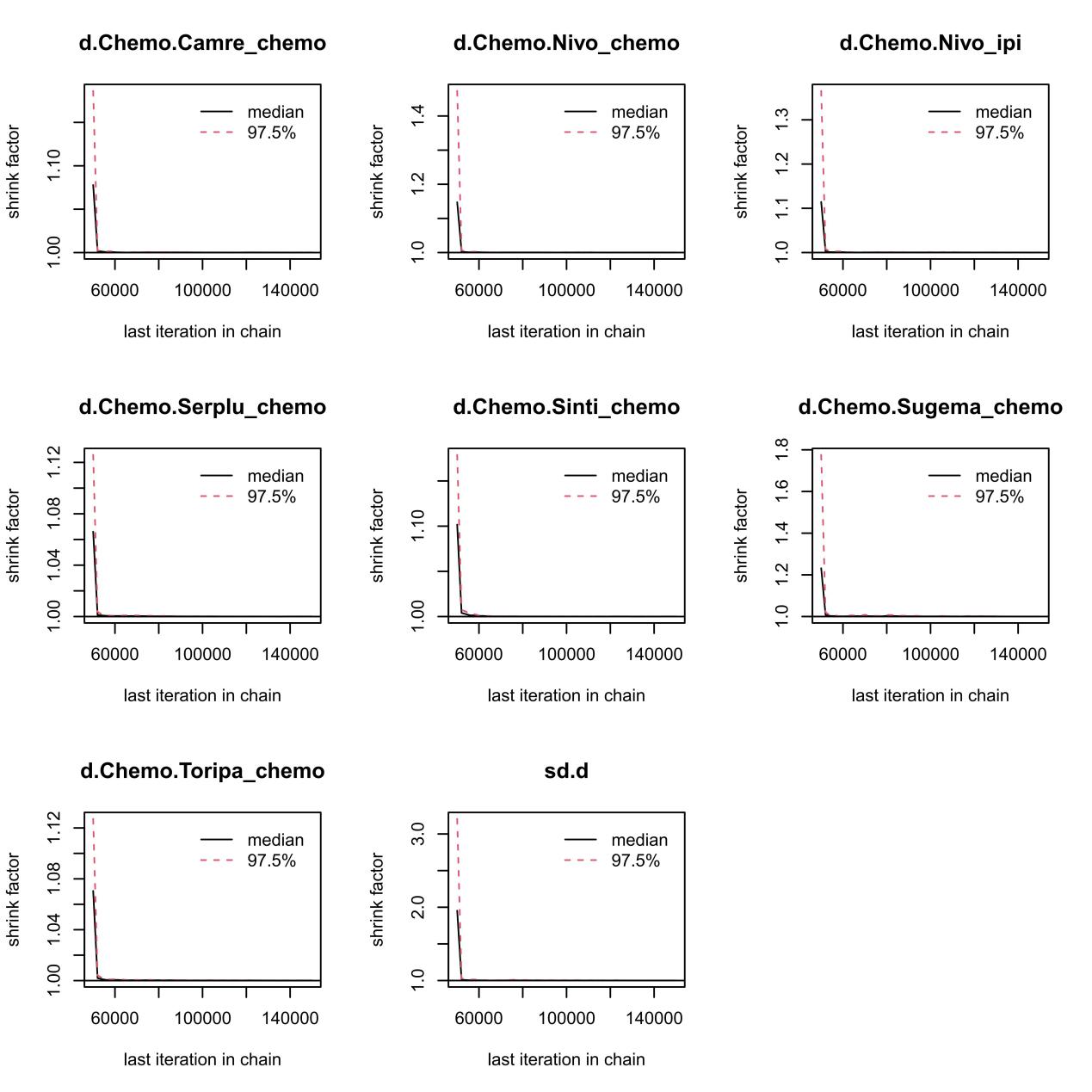
**

**Figure S18.Convergence Diagnostics of Bayesian Network Meta-Analysis for Immune-mediated rash in Advanced ESCC**

**
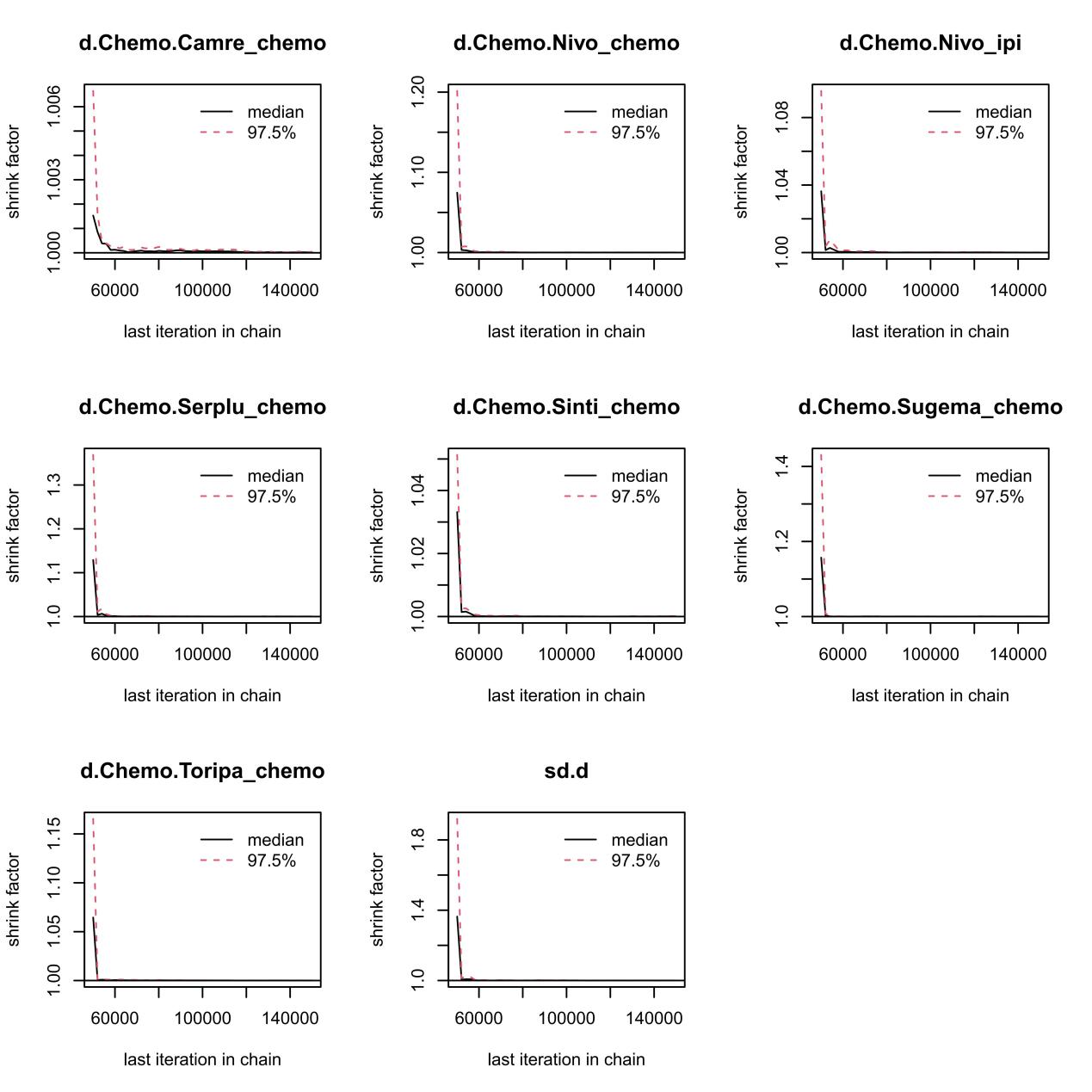
**

**Figure S19.Convergence Diagnostics of Bayesian Network Meta-Analysis for Immune-mediated hypothyroidism in Advanced ESCC**

**
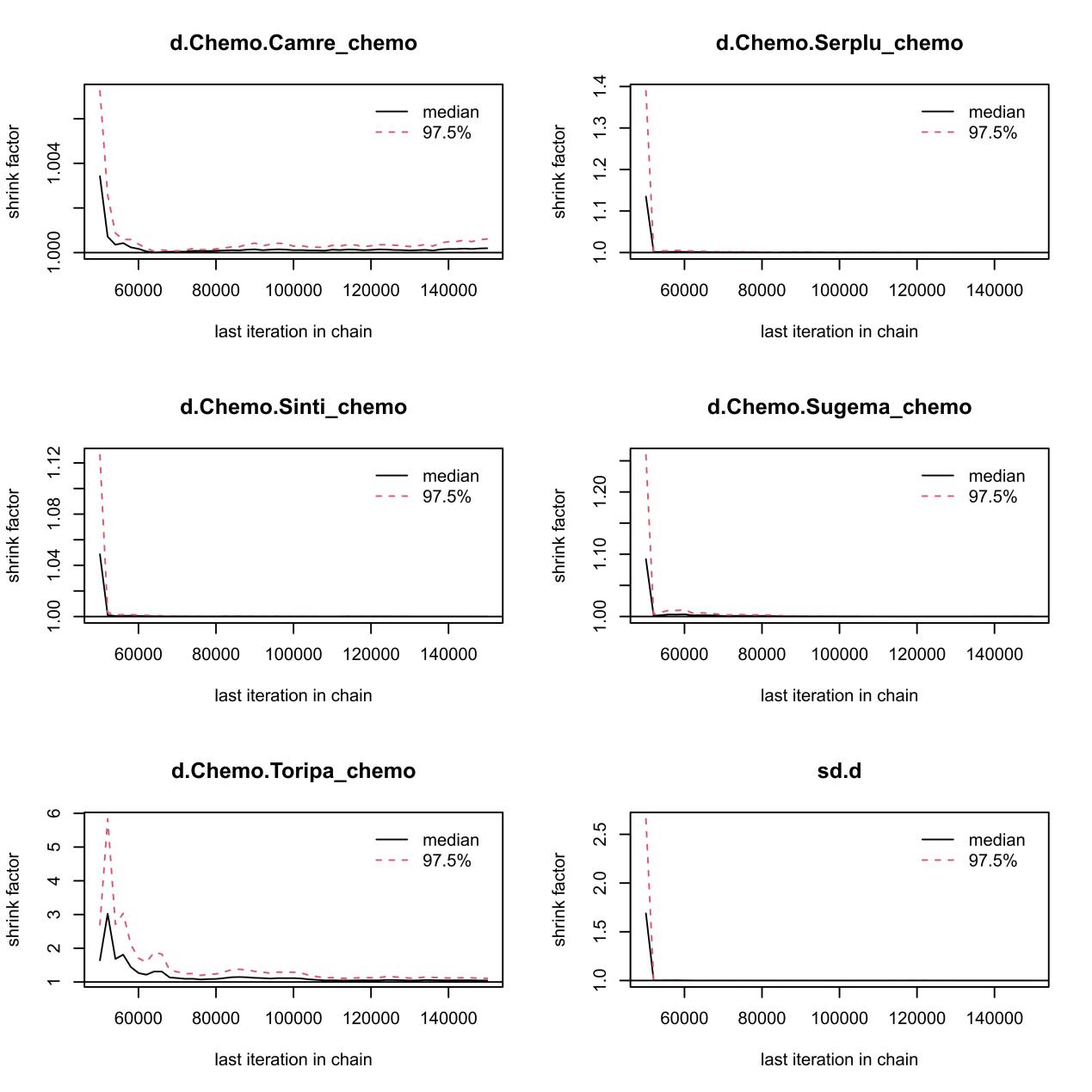
**

**Figure S20.Convergence Diagnostics of Bayesian Network Meta-Analysis for Immune-mediated hyperthyroidism in Advanced ESCC**

**
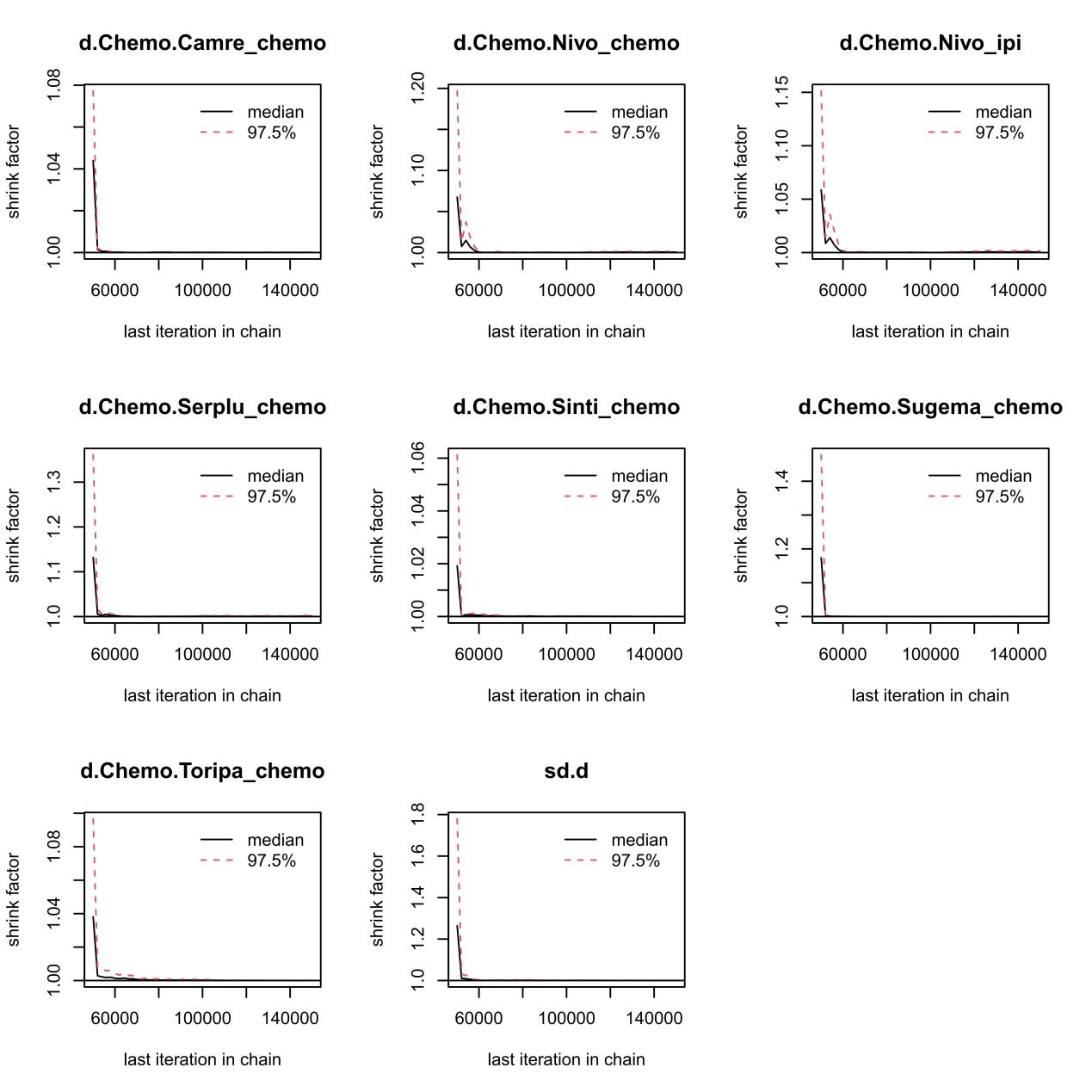
**

**Figure S21.Convergence Diagnostics of Bayesian Network Meta-Analysis for Immune-mediated Pneumonitis in Advanced ESCC**


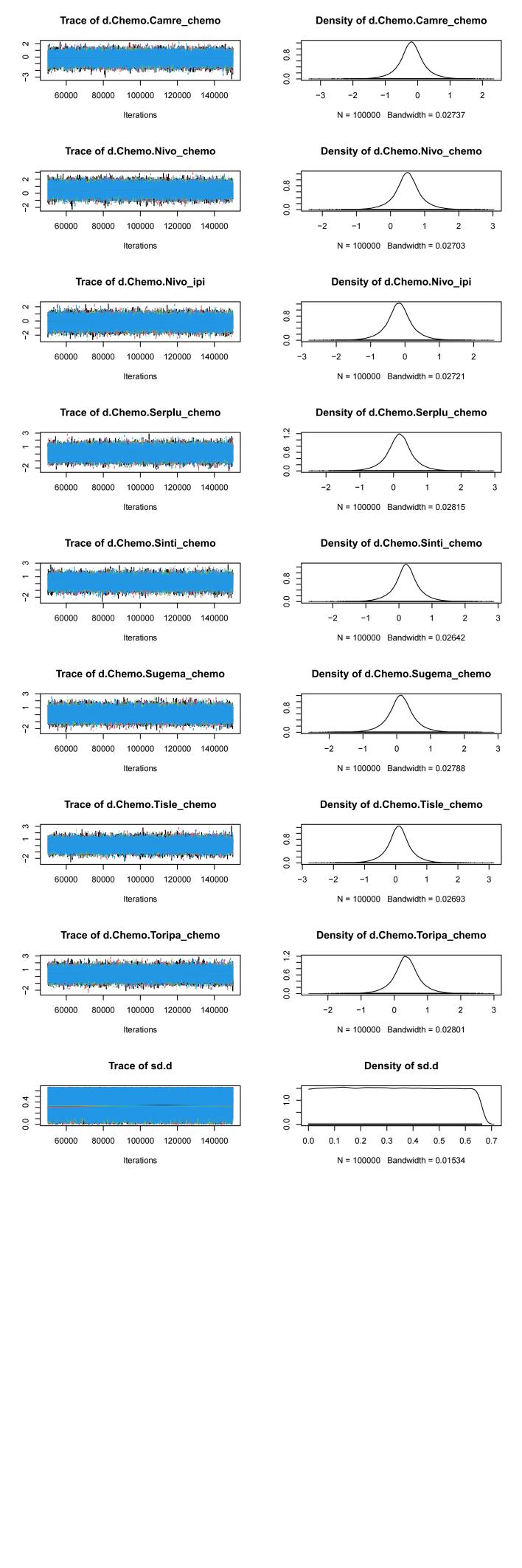


**Figure S22.Trace and Posterior Density Plots for Bayesian Network Meta-Analysis of Grade ≥3 trAEs in Advanced ESCC**

**
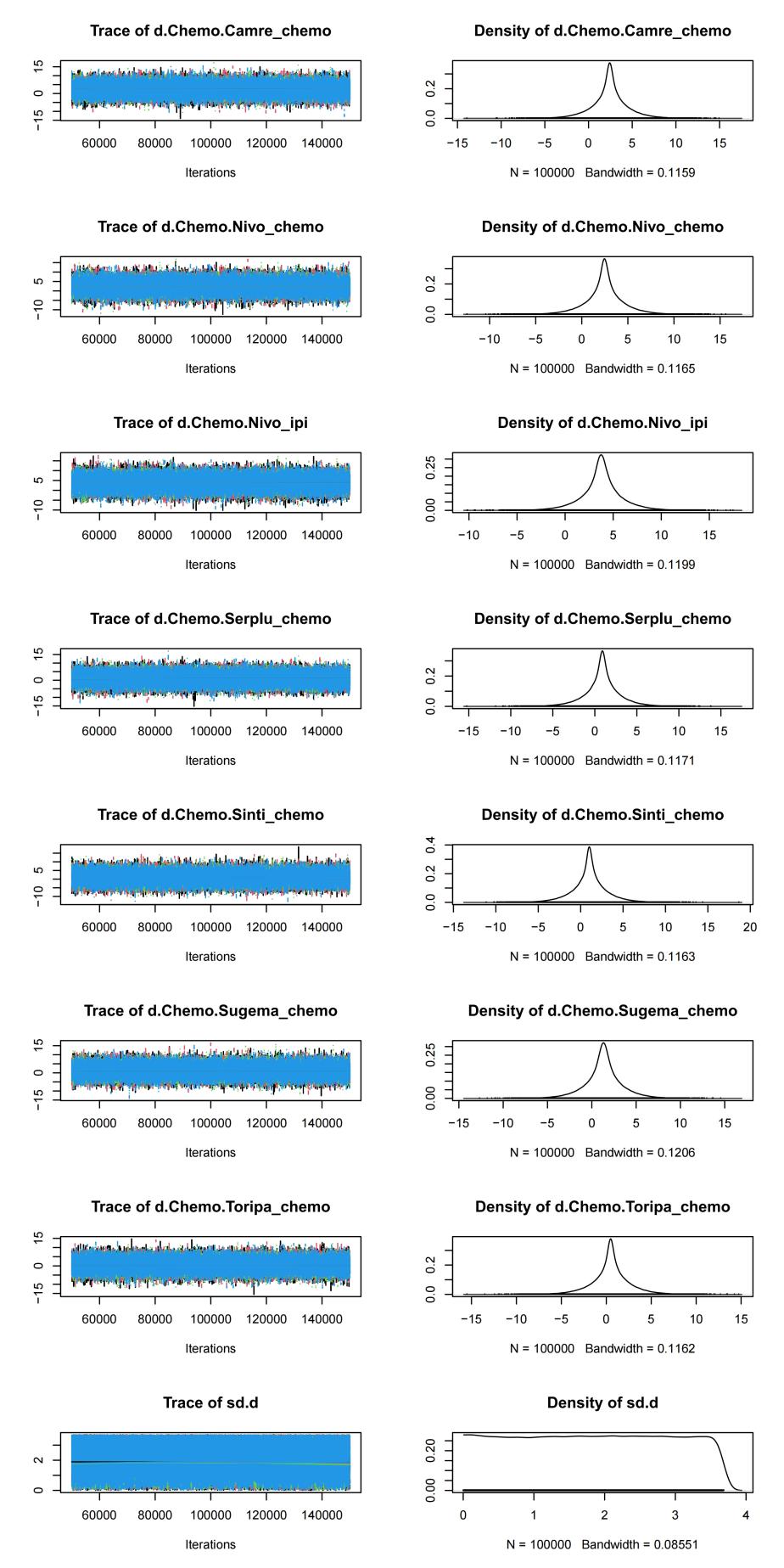
**

**Figure S23.Trace and Posterior Density Plots for Bayesian Network Meta-Analysis of Any irAEs in Advanced ESCC**

**
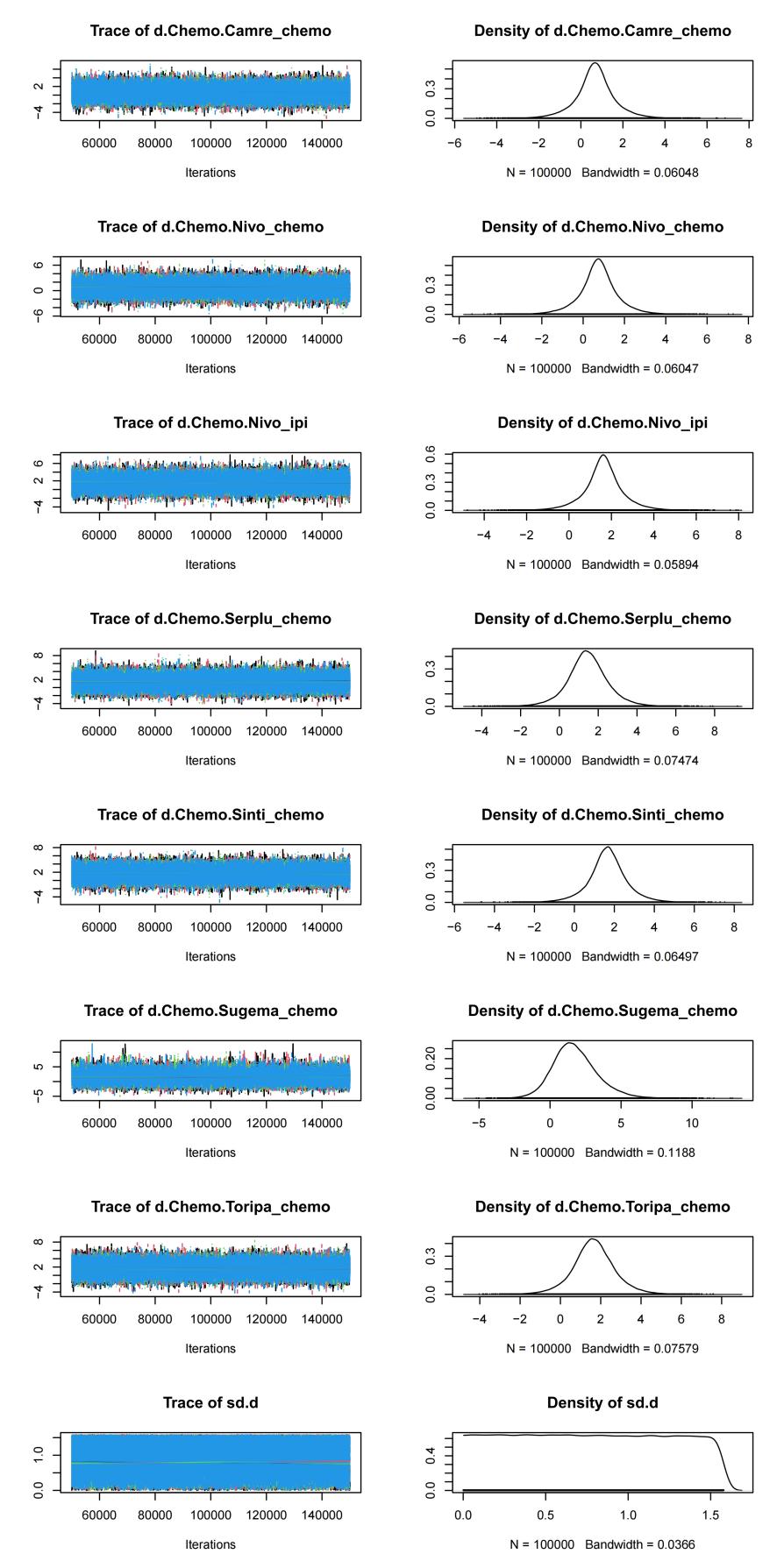
**

**Figure S24.Trace and Posterior Density Plots for Bayesian Network Meta-Analysis of Grade ≥3 irAEs in Advanced ESCC**

**
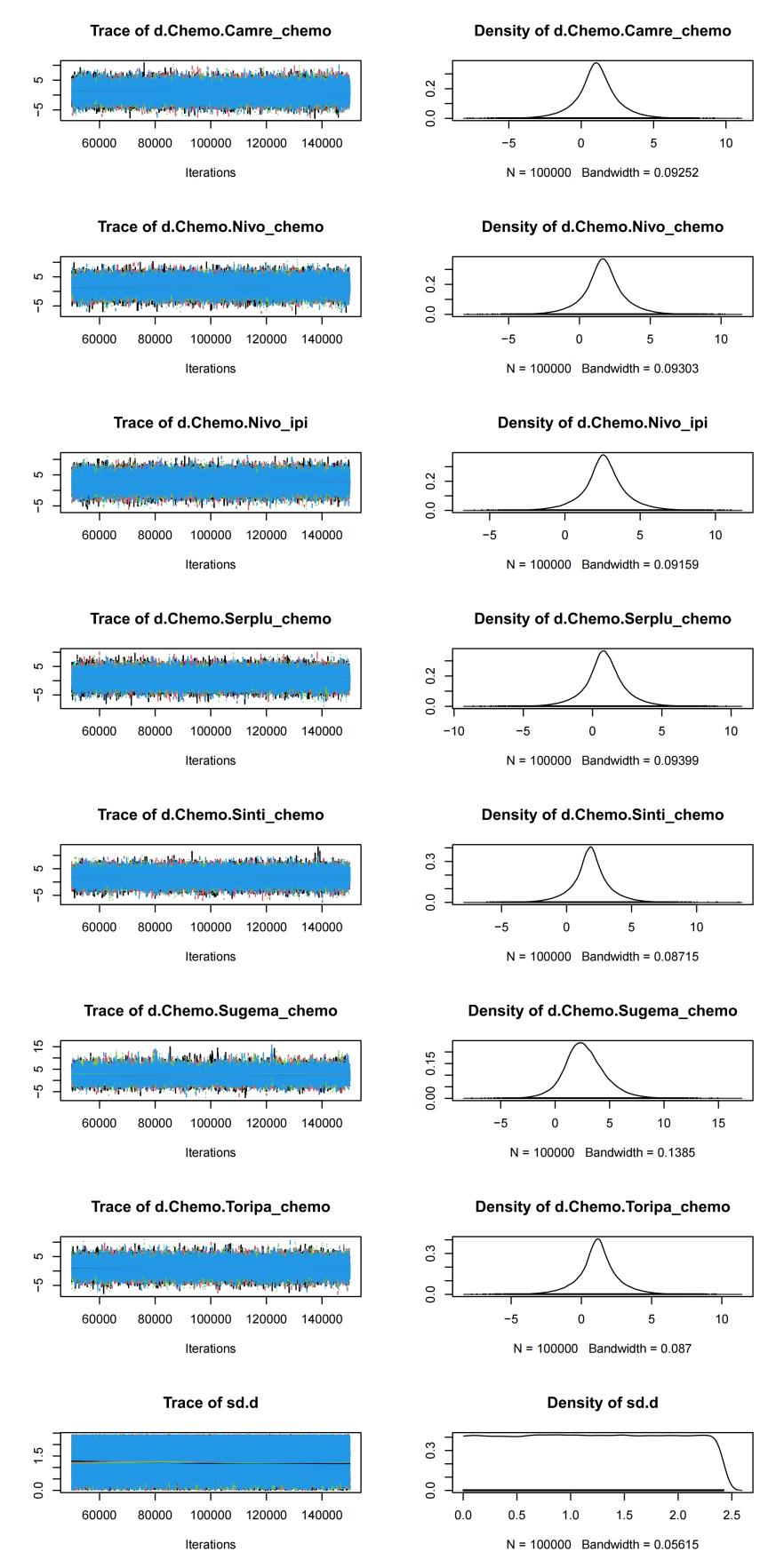
**

**Figure S25.Trace and Posterior Density Plots for Bayesian Network Meta-Analysis of Immune-mediated rash in Advanced ESCC**

**
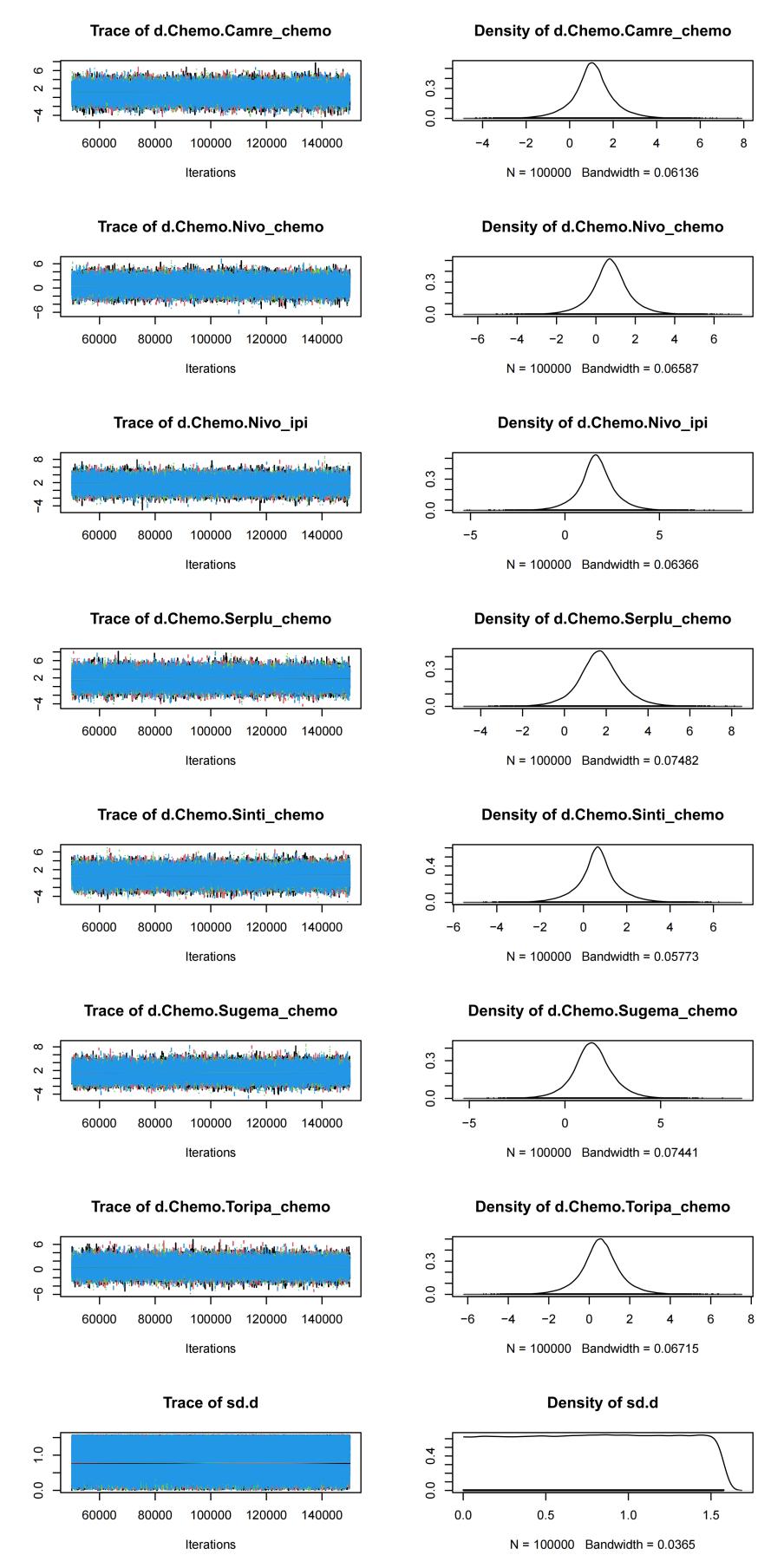
**

**Figure S26.Trace and Posterior Density Plots for Bayesian Network Meta-Analysis of Immune-mediated hypothyroidism in Advanced ESCC**

**
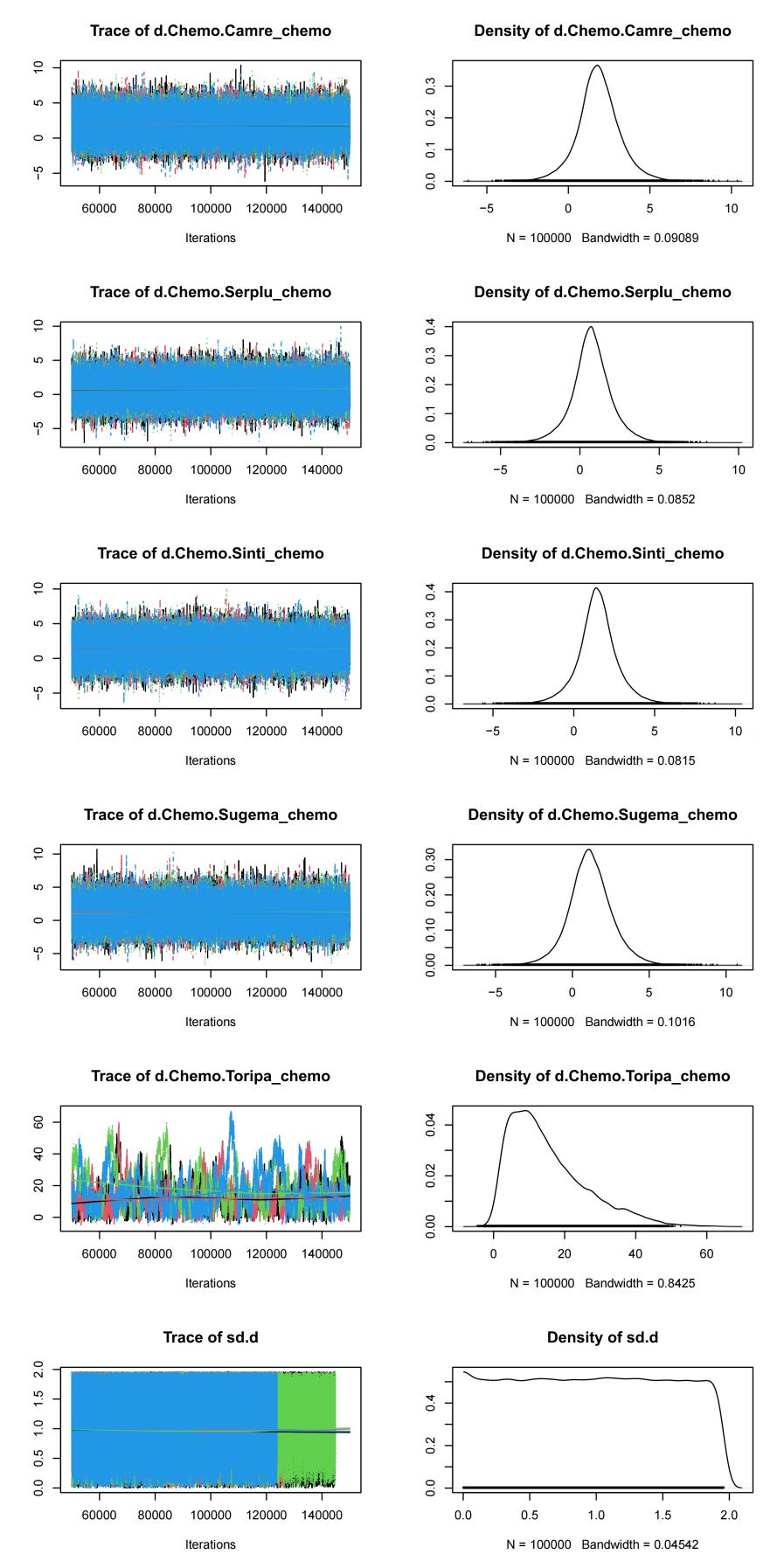
**

**Figure S27.Trace and Posterior Density Plots for Bayesian Network Meta-Analysis of Immune-mediated hyperthyroidism in Advanced ESCC**

**
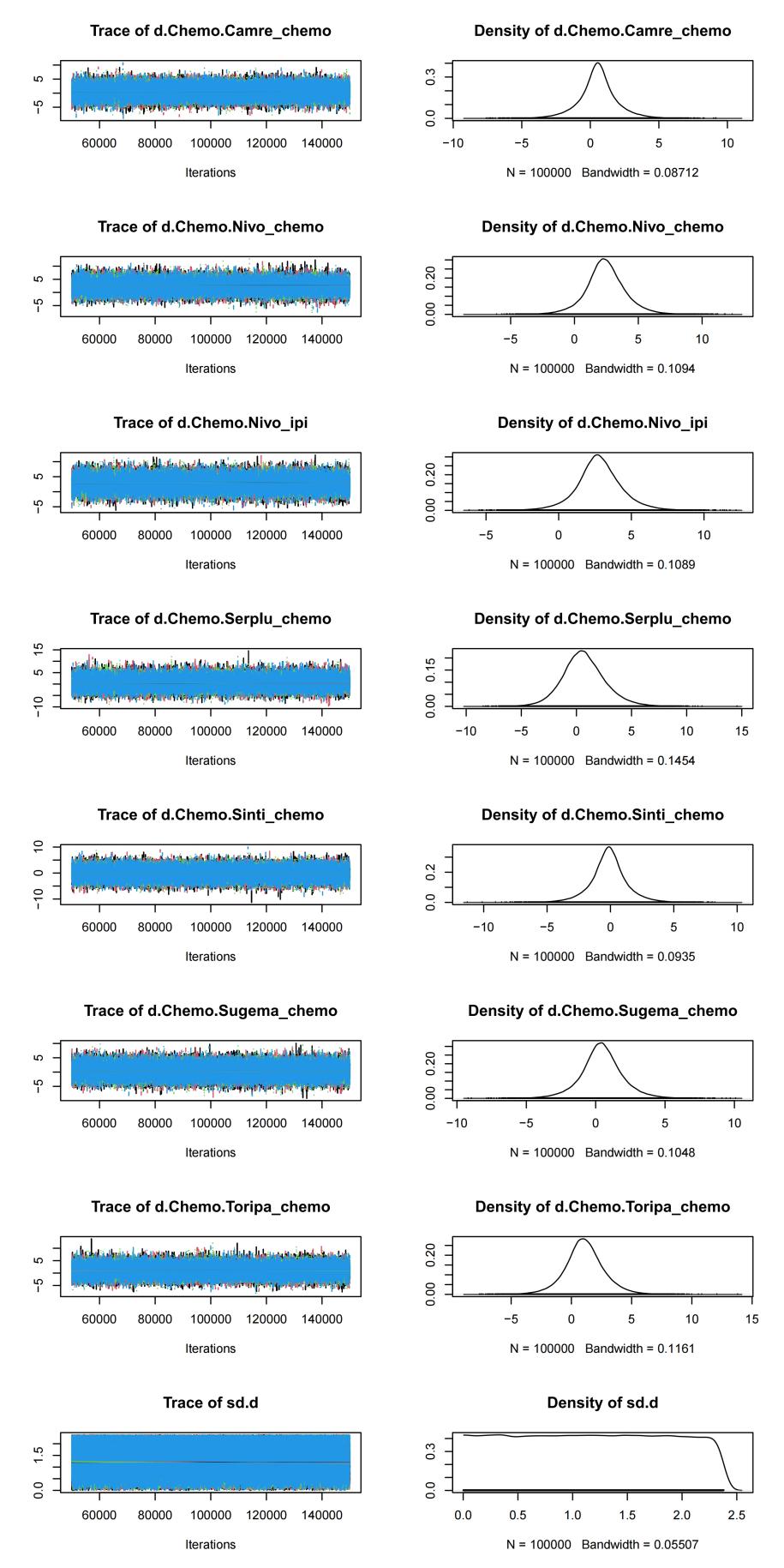
**

**Figure S28.Trace and Posterior Density Plots for Bayesian Network Meta-Analysis of Immune-mediated Pneumonitis in Advanced ESCC**


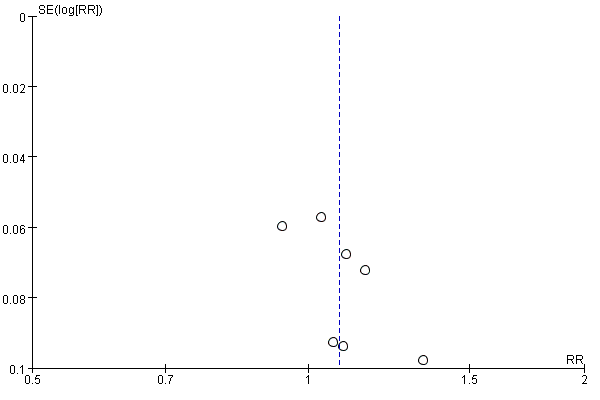


**Figure S29.Funnel Plot for Assessment of Publication Bias in Grade ≥3 trAEs Among Advanced ESCC Patients Receiving ICI-Based Combination Therapy Versus Chemotherapy**

**
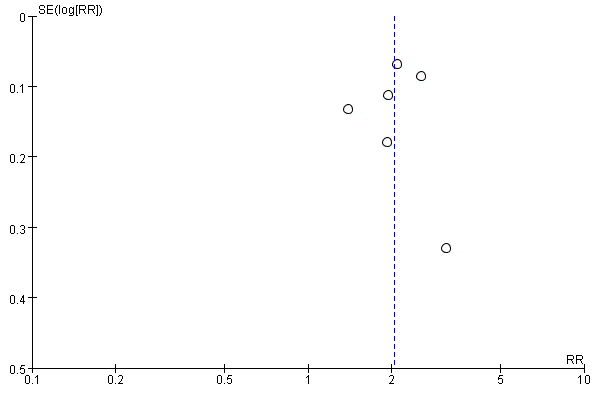
**

**Figure S30.Funnel Plot for Assessment of Publication Bias in Any irAEs Among Advanced ESCC Patients Receiving ICI-Based Combination Therapy Versus Chemotherapy**

**
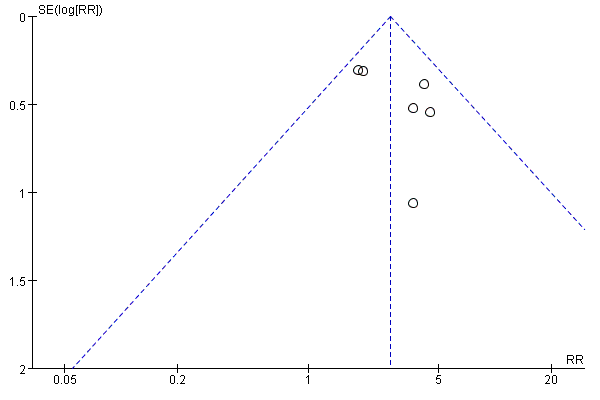
**

**Figure S31.Funnel Plot for Assessment of Publication Bias in Grade ≥3 irAEs Among Advanced ESCC Patients Receiving ICI-Based Combination Therapy Versus Chemotherapy**

**
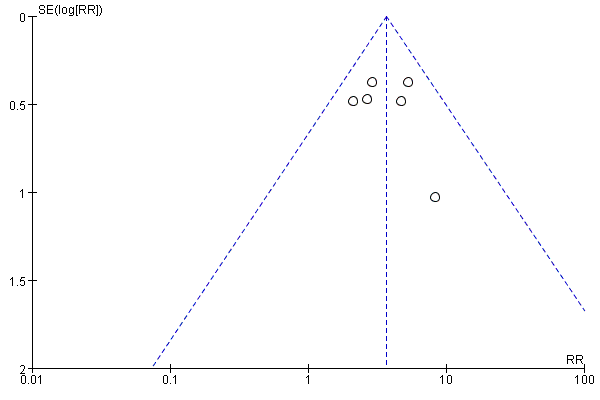
**

**Figure S32.Funnel Plot for Assessment of Publication Bias in Immune-mediated rash Among Advanced ESCC Patients Receiving ICI-Based Combination Therapy Versus Chemotherapy**

**
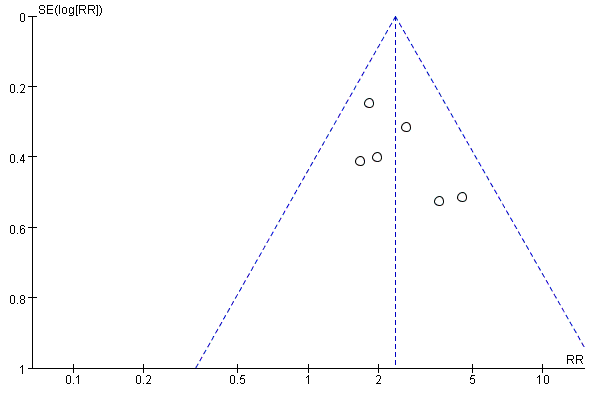
**

**Figure S33.Funnel Plot for Assessment of Publication Bias in Immune-mediated hypothyroidism**

**Among Advanced ESCC Patients Receiving ICI-Based Combination Therapy Versus Chemotherapy**
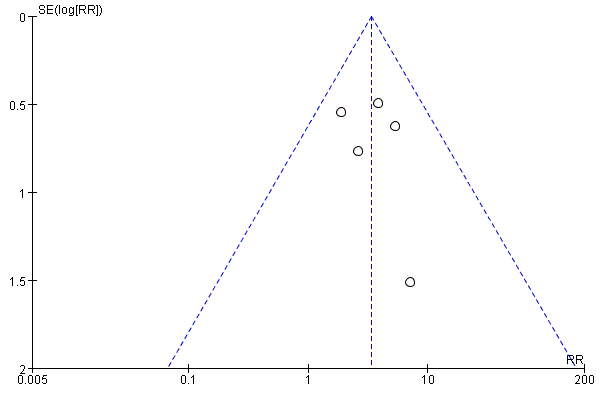


**Figure S34.Funnel Plot for Assessment of Publication Bias in Immune-mediated hyperthyroidism**

**Among Advanced ESCC Patients Receiving ICI-Based Combination Therapy Versus Chemotherapy**

**
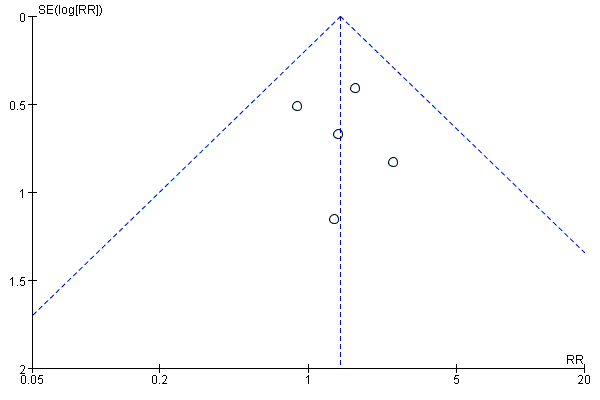
**

**Figure S35.Funnel Plot for Assessment of Publication Bias in Immune-mediated Pneumonitis Among Advanced ESCC Patients Receiving ICI-Based Combination Therapy Versus Chemotherapy**

Table S7.network meta-regression adjusted for chemotherapy regimen

| **Outcome** | **Covariate** | **Coefficient** | **Std. err.** | **P>z** | **[95% conf.** | **interval]** |
| --- | --- | --- | --- | --- | --- | --- |
| Grade ≥3 trAEs | chemotherapy regimen | 0.0820127 | 72.2798 | 0.999 | -141.6038 | 141.7278 |
| Any irAEs | chemotherapy regimen | -0.3377623 | 114.5746 | 0.998 | -224.8598 | 224.2642 |
| Grade ≥3 irAEs | chemotherapy regimen | 0.1943753 | 367.0525 | 1 | -719.2253 | 719.5941 |
| Immune-mediated  rash | chemotherapy regimen | 0.0429942 | 382.7713 | 1 | -750.177 | 750.259 |
| Immune-mediated  hypothyroidism | chemotherapy regimen | -0.1591802 | 312.2046 | 1 | -612.059 | 611.7607 |
| Immune-mediated  hyperthyroidism | chemotherapy regimen | -0.3881453 | 522.3665 | 0.999 | -1024.208 | 1023.431 |
| Immune-mediated  pneumonitis | chemotherapy regimen | 0.4874706 | 693.0811 | 0.999 | -1357.926 | 1358.901 |

Table S8.Definitions and Harmonization of Safety Endpoints Across Included RCTs

| **Study** | **CTCAE Version Used** | **Definition of Grade ≥3 trAEs** | **Ascertainment method for irAEs** | **Sponsor-defined criteria for immune-mediated AEs** | **organ-specific irAEs** |
| --- | --- | --- | --- | --- | --- |
| ORIENT-15 | CTCAE v5 | grade ≥3 treatment-related adverse events | investigator-reported | sponsor-defined immune-mediated AEs list | rash,hypothyroidism,hyperthyroidism,pneumonitis |
| JUPITER-06 | CTCAE v5 | grade ≥3 treatment-related adverse events | investigator-reported | sponsor-predefined immune-related MedDRA terms | rash,hypothyroidism,hyperthyroidism,pneumonitis |
| ESCORT-1st | CTCAE v4.03 | grade ≥3 treatment-related adverse events | investigator-reported | sponsor-predefined immune-related MedDRA terms | rash,hypothyroidism,hyperthyroidism,pneumonitis |
| Checkmate 648 | CTCAE v4.0 | grade ≥3 treatment-related adverse events | investigator-reported with post-hoc adjudication | sponsor-defined immune-mediated AEs list | rash,hypothyroidism |
| ASTRUM-007 | CTCAE v4.03 | grade ≥3 treatment-related adverse events | investigator-reported | based on a sponsor pre-defined MedDRA immune-mediated AE list | rash,hypothyroidism |
| GEMSTONE-304 | CTCAE v5 | grade ≥3 treatment-related adverse events | investigator-reported | sponsor-defined immune-mediated AEs list | rash,hypothyroidism,hyperthyroidism,pneumonitis |
| RATIONALE-306 | CTCAE v4.03 | grade ≥3 treatment-related adverse events | - | - | - |

Table S9.Raw Event Counts for Grade ≥3 trAEs, Any-Grade irAEs, and Grade ≥3 irAEs Across Included Trials

| **Study** | **Grade ≥3 trAEs** | | | | **Any irAEs** | | | | **Grade ≥3 irAEs** | | | |
| --- | --- | --- | --- | --- | --- | --- | --- | --- | --- | --- | --- | --- |
|  | **Events (experimental)** | **Total (experimental)** | **Events  (control)** | **Total  (control)** | **Events (experimental)** | **Total (experimental)** | **Events  (control)** | **Total  (control)** | **Events (experimental)** | **Total (experimental)** | **Events  (control)** | **Total  (control)** |
| ORIENT-15 | 196 | 327 | 181 | 332 | 155 | 327 | 81 | 332 | 33 | 327 | 8 | 332 |
| JUPITER-06 | 166 | 257 | 144 | 257 | 95 | 257 | 68 | 257 | 18 | 257 | 4 | 257 |
| ESCORT-1st | 189 | 298 | 201 | 297 | 252 | 298 | 98 | 297 | 28 | 298 | 15 | 297 |
| Checkmate 648 | 147/102 | 310/322 | 108 | 304 | 278/312 | 310/322 | 130 | 304 | 28/62 | 310/322 | 14 | 304 |
| ASTRUM-007 | 201 | 382 | 81 | 168 | 132 | 382 | 30 | 168 | 33 | 382 | 4 | 168 |
| GEMSTONE-304 | 181 | 352 | 88 | 182 | 61 | 352 | 10 | 182 | 7 | 352 | 1 | 182 |
| RATIONALE-306 | 216 | 324 | 207 | 321 |  |  |  |  |  |  |  |  |

Table S10.Raw Event Counts for Organ-Specific Immune-Related Adverse Events Across Included Trials

| **Study** | **immune-mediated rash** | | | | **immune-mediated hypothyroidism** | | | | **immune-mediated hyperthyroidism** | | | | **immune-mediated pneumonitis** | | | |
| --- | --- | --- | --- | --- | --- | --- | --- | --- | --- | --- | --- | --- | --- | --- | --- | --- |
|  | **Events (experimental)** | **Total (experimental)** | **Events  (control)** | **Total  (control)** | **Events (experimental)** | **Total (experimental)** | **Events  (control)** | **Total  (control)** | **Events (experimental)** | **Total (experimental)** | **Events  (control)** | **Total  (control)** | **Events (experimental)** | **Total (experimental)** | **Events  (control)** | **Total  (control)** |
| ORIENT-15 | 42 | 327 | 8 | 332 | 41 | 327 | 23 | 332 | 19 | 327 | 5 | 332 | 7 | 327 | 8 | 332 |
| JUPITER-06 | 26 | 257 | 9 | 257 | 15 | 257 | 9 | 257 | 3 | 257 | 0.5 | 258 | 5 | 257 | 2 | 257 |
| ESCORT-1st | 16 | 298 | 6 | 297 | 34 | 298 | 13 | 297 | 16 | 298 | 3 | 297 | 15 | 298 | 9 | 297 |
| Checkmate 648 | 24/55 | 310/322 | 5 | 304 | 18/43 | 310/322 | 9 | 304 | - | - | - | - | - | - | - | - |
| ASTRUM-007 | 24 | 382 | 5 | 168 | 41 | 382 | 4 | 168 | 17 | 382 | 4 | 168 | 3 | 382 | 1 | 168 |
| GEMSTONE-304 | 16 | 352 | 1 | 182 | 28 | 352 | 4 | 182 | 10 | 352 | 2 | 182 | 8 | 352 | 3 | 182 |
